# Association between cardiovascular disease- and inflammation-related serum biomarkers and poor lung function in elderly

**DOI:** 10.1186/s12014-021-09329-7

**Published:** 2021-09-28

**Authors:** K. Egervall, A. Rosso, S. Elmståhl

**Affiliations:** Division of Geriatric Medicine, Department of Clinical Sciences in Malmö, Lund University, Malmö, Sweden

**Keywords:** Lung function decline, Proteomics, Cardiovascular disease, Inflammation

## Abstract

**Background:**

Cardiovascular disease (CVD) is a common comorbidity in chronic obstructive pulmonary disease (COPD) and reduced lung function is an important risk factor for CVD and CVD-related death. However, the mechanisms behind the increased risk for CVD in COPD patients are not fully understood.

**Methods:**

We examined the association between CVD- and inflammation-related serum biomarkers, and pulmonary function in a geriatric population. 266 biomarkers related to CVD and inflammation were analyzed in blood samples from 611 subjects aged 66–86 years who participated in the Good Aging in Skåne study. Serum levels were assessed by a proximity extension assay. Pulmonary function was measured using the lower limit of normality (LLN) spirometry criteria, i.e., forced expiratory volume in 1 s (FEV1)/forced vital capacity (FVC)  <  LLN. Logistic regression models were implemented and multiple comparisons were accounted for.

**Results:**

10.3% of the study participants fulfilled pulmonary function decline criteria according to LLN. Out of the 266 biomarkers, only plasminogen activator, urokinase receptor (PLAUR) was statistically significantly associated with decreased pulmonary function. We could not find a statistically significant association between pulmonary function decline and other biomarkers previously linked to COPD, such as interleukin 6, tumor necrosis factor and surfactant protein D.

**Conclusion:**

We found that serum levels of PLAUR are associated with pulmonary function decline in older adults. PLAUR is activated following inflammation and promotes matrix metallopeptidase (MMP) activation and extracellular matrix (ECM) degradation. This implies that PLAUR could play a role in the early phase of COPD pathogenesis.

**Supplementary Information:**

The online version contains supplementary material available at 10.1186/s12014-021-09329-7.

## Background

Chronic obstructive pulmonary disease (COPD), characterized by reduced lung function, is a common cause of death; World Health Organization (WHO) reports COPD to cause 5% of deaths globally in 2015 [1]. Clinically, COPD is typically diagnosed using spirometry. The Global Initiative for Chronic Obstructive Lung Disease (GOLD) recommends using the ratio of forced expiratory volume in 1 s (FEV1) to forced vital capacity (FVC) to assess whether the patients suffer of airflow limitation. According to the GOLD criterium, values of FEV1/FVC less than 70% after bronchodilator administration are an indication of airflow obstruction [2]. COPD diagnosis as defined by GOLD also requires respiratory symptoms [2]. Normal ageing causes a natural decline in the lung function which varies with sex and ethnicity [3]. Therefore, a fixed ratio tends to overestimate COPD prevalence in the elderly [4]. To overcome these limitations, the European Respiratory Society (ERS) instead recommends comparing the FEV1/FVC to population-based reference values [5]. According to this definition, individuals with potential airflow obstruction are identified as those with FEV1/FVC below the lower limit of normal (LLN; below the fifth percentile adjusted for age, sex, height, and ethnic group) [4]. In this study, we use the terms pulmonary function decline, lung function decline, and airflow obstruction as an estimate of COPD as we primarily assessed spirometry results and proteomics and not symptoms.

COPD prevalence differs among studies, on the basis of definition criteria, age and regional differences [6, 7]. In an Austrian sample of 1258 Salzburg County inhabitants aged  ≥  40 years the prevalence of irreversible airflow obstruction defined by the GOLD criteria was estimated to 26.1% [8]. A Dutch study presented an LLN-based COPD prevalence of 19% in a population-based sample of adults aged  ≥  40 years [9]. In the Swedish BOLD study, conducted on 548 urban individuals aged 40 years or more [10], COPD prevalence using LLN criteria was calculated to be 10%, compared to 16.2% using GOLD criteria. Backman et al. [11] found an LLN-based COPD prevalence of 4.9% in a Swedish population aged 21–78 years, n  =  1839. The OLIN studies showed a linear increase in COPD prevalence from 45 years of age in smokers in the north of Sweden [12]. Luoto et al. [13] showed a linear effect of age on incidence for airflow limitation for fixed-ratio criteria but not for LLN criteria.

Cardiovascular disease (CVD) is a common comorbidity in COPD and reduced lung function is an important risk factor for CVD and CVD-related death [14]. However, the mechanisms behind the increased risk for CVD in COPD patients are not fully understood. Previous research has identified inflammatory proteins related to systemic inflammation that could distinguish COPD patients from healthy control subjects [15, 16]. Briefly, inflammation is a pathological process mediated by cytologic or chemical reactions [17], and several conditions can lead to increased inflammatory biomarkers in COPD patients. Local inflammation in the airways can cause inflammatory molecules, such as cytokines and chemokines, to be released into the blood stream, leading to systemic inflammation [14]. Repeated exposure of cigarette smoke and air pollution causes chronic inflammation in central and peripheral airways, lung parenchyma and blood vessels in the lungs [18–21]. Furthermore, parallel processes such as atherosclerosis, heart failure and diabetes can lead to increased systemic inflammation in COPD patients [14].

Proteomics enables simultaneous analysis of the content of several biomarkers in various fluids. It has previously been used to identify potential biomarkers for the pathogenesis of various diseases, including COPD [22, 23]. By identifying CVD and inflammatory biomarkers and investigating their association with lung function, information about airflow obstruction and its association with CVD and systemic inflammation might unveil. This knowledge can provide a deeper understanding of the mechanisms behind CVD, inflammation, and pathogenesis of airflow obstruction. In addition, the presence of biomarkers can potentially improve diagnostic. COPD diagnosis currently requires a subject to be physically and cognitively fit to provide an acceptable spirometry, which hampers diagnosis of the frailest subjects. Serum biomarkers could potentially serve as a diagnostic tool in this group.

In the current study, we investigate the association between CVD, inflammation, and lung function in an elderly population. We analyse the association of 266 serum potential biomarkers related to CVD and inflammation with lung function.

## Materials and methods

### Study design and participants

The current study is a part of the general longitudinal population study Good Aging in Skåne (Gott Åldrande i Skåne, GÅS) that started in 2001 [24]. The GÅS project comprises three waves recruited 2001–2004 (n  =  2931, Wave 1), 2007–2009 (n  =  1523, Wave 2) and 2013–2015 (n  =  1350, Wave 3), with a participation rate between 60 and 70%. Subjects aged from 60 to 93 years were randomly invited from the Swedish population register [25]. After inclusion, subjects were examined and questioned by a physician, a nurse, and a medical secretary at one out of four research test centres in Skåne. Examination included medical history, electrocardiography, blood samples, spirometry and cognitive tests. Study participants attended follow up visits every third year (subjects aged  ≥  78 years) or every sixth year (subjects aged  <  78 years).

632 blood samples from subjects consecutively examined during 2015–2018 were retrieved from the GÅS study. At that point in time, the prevalence of airflow obstruction in this GÅS population was not fully established. Therefore, to assure that at least some subjects in the study population suffered from airflow obstruction, 72 subjects known to fulfil the COPD criteria according to GOLD were also included. In total, 704 blood samples were analysed.

### Proteomic analysis

The blood samples were drawn and immediately stored at -80 degree Celsius. Proteomic analysis and quality control (QC) was performed by Olink, Olink Proteomics AB, Uppsala, Sweden, at Clinical Biomarkers Facility, Science for Life Laboratory, Uppsala University, Sweden [25] in 2019. Briefly, the Olink Proseek Multiplex CVD II, CVD III, and Inflammation kits, measuring 266 CVD- and inflammation-related biomarkers in plasma were used [26]. Ten of the biomarkers overlapped and belonged to more than one panel. The analysis was performed using the proximity extension assay (PEA) [27]. Quality control (QC) was performed by adding four internal controls to each sample and external controls in every analysis. The quality control process was performed in two steps. First each sample plate was evaluated on the standard deviation of the internal controls. This should be below 0.2 NPX. Only data from sample plate that pass this quality control are used in the second step of the quality control process. The quality of each sample was further assessed by evaluating the deviation from the median value of the controls for each individual sample. Samples that deviate less than 0.3 NPX from the median pass the quality control. Samples that did not pass the QC were excluded from the analyses. Values below the limit of detection (LOD) were included in the primary analysis. 654 out the 704 participants’ blood samples passed the Olink QC analysis.

### Spirometry

Spirometries were performed using the Vitalograph 2120 electronic flow volume spirometer using Spirotrac IV software (Vitalograph Ltd., Buckingham, UK). The spirometry, including daily calibration, was performed according to American Thoracic Society (ATS) guidelines using bronchodilators by one and the same registered nurse [28]. Up to a maximum of 8 consecutive breathing maneuvers were performed with the goal of completing at least three acceptable curves. Reproducibility criteria was used as an indication of whether more maneuvers were necessary. To avoid exclusion of very frail individuals, only one acceptable manoeuvre was required [29]. Spirometry and blood samples were collected on the same visit.

### Definition of airflow obstruction

We defined airflow obstruction using the LLN criterion, as described in the 2012 Global Lung Function Initiative reference equations for the predicted FEV1/FVC ratios [30, 31]. We choose the LLN criterion because it has a higher specificity compared to the GOLD criterium in the elderly population [32].

### Statistics

The strength of the association between the biomarkers and subjects with air flow obstruction was estimated using a logistic regression model. A raw model was implemented to assess which protein biomarkers are associated with poor lung capacity. Each protein panel included 93 proteins which were analyzed separately. To acknowledge that samples from the same participants were used to calculate 93 different logistic models (one for each protein) and that protein levels may be correlated, the Yekutieli method [33] was used to keep the false positive discovery (FDR) rate at 5%. This method was preferred over the Benjamini and Hockberg method since the Yekutieli method is expected to control the FDR regardless of the correlation among the p values [34]. Age, sex, and smoking affect the airflow and may also affect the protein concentration levels. To enhance the interpretability of our results, a logistic model including these factors as covariates was applied only to those protein biomarkers that were statistically significant in the crude logistic model. A likelihood ratio test was used to evaluate the difference between the crude and adjusted models.

To assess the impact of the inclusion of values below LOD, the statistical analyses were repeated excluding those values.

The statistical analyses were performed using Stata IC 14.2 software (StataCorp LLC, 4905 Lakeway Drive, College Station, TX 77845, USA). The calculations of the p values were performed using the package q-values [34].

## Results

### Patient characteristics

**Fig. 1:**
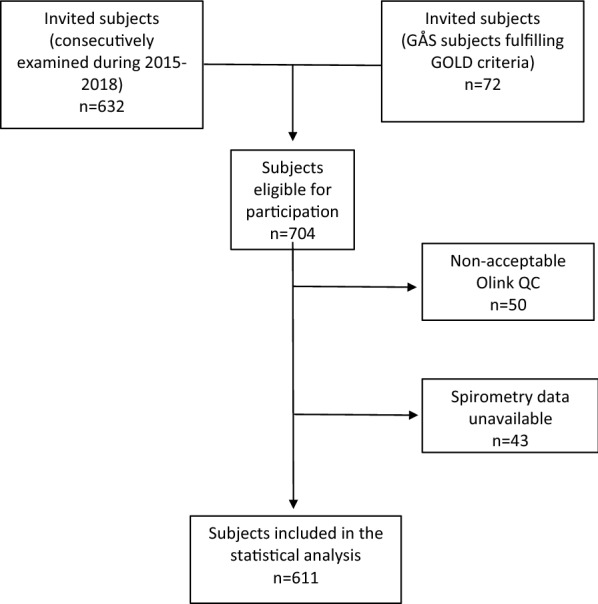
Number of participants

The patient flow chart is shown in Fig. 1. In total, 704 subjects were included in the study. Of those 611 subjects had completed spirometry and had blood samples that passed the Olink quality control procedure. Baseline characteristics are presented in Table 1. 10.3% had an FEV1/FVC ratio below LLN, i.e., airflow obstruction according to LLN criteria. Notably, only 60.3% of subjects fulfilling the LLN airflow obstruction-criteria were diagnosed with COPD according to medical records. 6.2% of subjects with normal pulmonary function were diagnosed with COPD. 58.9% of the study population were current or former smokers.

**Table 1:** Demographic characteristics

	FEV1/FVC ≥ LLN	FEV1/FVC < LLN	Missing FEV1/FVC
Sex			
Male (n, %)	256 (46.7)	21 (33.3)	18 (41.9)
Female (n, %)	292 (53.3)	42 (66.7)	25 (58.1)
Age			
N	548	63	43
Median	77	78	72
Min.	66	66	60
Max.	86	85	85
BMI			
N	547	62	39
Median	26.7	25.0	26.3
Min.	17.9	16.7	18.6
Max.	44.1	36.9	38.7
Walking test 15 m (sec)			
N	539	60	38
Median	9.4	10.7	10.5
Min.	6.2	6.6	7.3
Max.	19.0	21.9	23.2
Smoking status			
Smoker (n, %)	51 (9.3)	18 (28.6)	3 (7.0)
Former smoker (n, %)	258 (47.1)	33 (52.4)	25 (58.1)
Never smoker (n, %)	237 (43.2)	12 (19.0)	15 (34.9)
Missing (n, %)	2 (0.4)	0 (0.0)	0 (0.0)
Cardiovascular disease			
Angina pectoris			
No (n, %)	502 (91.6)	57 (90.5)	38 (88.4)
Yes (n, %)	46 (8.4)	6 (9.5)	5 (11.6)
Ischaemic stroke			
No (n, %)	490 (89.4)	55 (87.3)	36 (83.7)
Yes (n, %)	58 (10.6)	8 (12.7)	7 (16.3)
TIA			
No (n, %)	519 (94.7)	60 (95.2)	41 (95.3)
Yes (n, %)	29 (5.3)	2 (3.2)	2 (4.7)
Missing (n, %)	0 (0.0)	1 (1.6)	0 (0.0)
Cerebral hemorrhage			
No (n, %)	546 (99.6)	62 (98.4)	43 (100.0)
Yes (n, %)	2 (0.4)	1 (1.6)	0 (0.0)
Hypertension			
No (n, %)	272 (49.6)	26 (41.3)	22 (51.2)
Yes (n, %)	276 (50.4)	37 (58.7)	21 (48.8)
COPD			
No (n, %)	514 (93.8)	25 (39.7)	39 (90.7)
Yes (n, %)	34 (6.2)	38 (60.3)	4 (9.3)
Diabetes (type I and type II)			
No (n, %)	474 (86.5)	55 (87.3)	38 (88.4)
Yes (n, %)	74 (13.5)	8 (12.7)	5 (11.6)
FEV1			
N	548	63	43
Median	2.2	1.4	NA
Min.	0.8	0.5	NA
Max.	4.5	2.7	NA
FVC			
N	548	63	43
Median	2.9	2.6	NA
Min.	0.8	1.1	NA
Max.	5.8	4.8	NA
FEV1/FVC			
N	548	63	43
Median	0.8	0.5	NA
Min.	0.6	0.3	NA
Max.	1.0	0.7	NA

*TIA* transient ischemic attack; *COPD* chronic obstructive pulmonary disease; *BMI* body mass index; *FEV* forced expiratory volume; *FVC* forced vital capacity

### CVD and inflammation biomarker profile

**Fig. 2:**
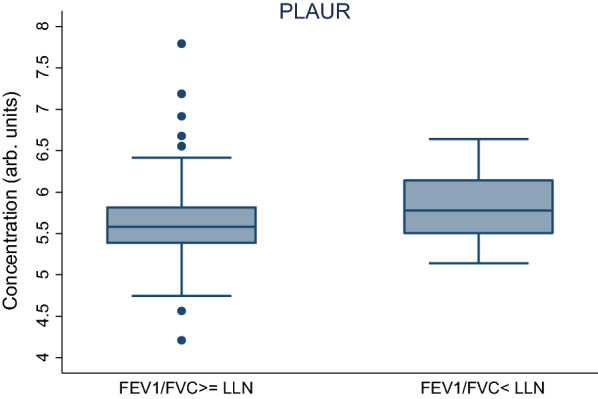
Concentration of PLAUR for participants with and without FEV1/FVC below the lower limit of normality. *FEV1* force expiratory volume in the first second. *FVC* forced vital capacity

We investigated association in all proteins included in the panels, and no results were discarded. Only one of the 266 analysed biomarkers, plasminogen activator, urokinase receptor (PLAUR), was significantly associated with pulmonary function decline according to LLN (FDR  <  5%, P  <  3.52  ×  10^–5^). The concentration levels for PLAUR are shown in Fig. 2. It is seen that subjects with poor lung capacity tend to have higher concentration levels of PLAUR. Odds ratio for PLAUR was 4.37 95% CI (2.17–8.79) (see Table 2). This indicates that subjects with decreased lung function were more likely to have increased levels of PLAUR than subjects without lung function decrease. The results for the adjusted logistic model including age, sex and smoking status are shown in Table 2. Also in this model, participants with poor lung capacity have higher concentration levels of PLAUR. The likelihood ratio test suggested that the adjusted model improved the model fitting compared to the crude model (p value 0.0014). The results of the crude logistic model for the other proteins are reported in the Additional file 1 (see Table S1).

**Table 2:** Logistic regression to assess the association between the biomarker soluble urokinase plasminogen activator surface receptor (U-PAR) and poor pulmonary function (FEV1/FVC  <  LLN)

	Odds ratio	95 % CI	Raw p-value
Raw model			
Concentration U-PAR (arbt. units)	4.37	2.17–8.79	< 0.0001
Adjusted model			
Concentration U-PAR (arbt. units)	3.6	1.71–7.57	0.001
Age (years)	1.03	0.96–1.10	0.356
Sex (female vs male)	0.51	0.29–0.90	0.02
Smoking (smoker vs never smoker)	3.05	1.57–5.92	0.001

The Yekutieli method was used to control the false discovery rate at 5%. Only statistically significant results are shown

### Sensitivity analyses

The statistical analyses were repeated excluding all values below the limit of detection. There were 2 protein biomarkers from the inflammation panel (IL2 and IFNG) that were completely excluded from the analyses since none or very few values remained above the LOD. The results are shown in the Additional file 1 (Table S2) and were consistent with those presented in the primary analysis.

## Discussion

In this study, we investigate how CVD- and inflammation-related serum biomarkers are related to poor lung function in the elderly. We found that subjects with limited airflow tend to have higher concentration of PLAUR. Previous research has shown an association between increased levels of serum PLAUR and COPD in middle-aged patients with moderate COPD [35–38]. The mechanism behind this association is not fully understood. Upon activation by the serine protease plasminogen activator, urokinase (PLAU), following inflammation, infection or malignancy, PLAUR activates plasminogen into plasmin, promoting inflammatory cell migration and activation, extracellular matrix (ECM) degradation and matrix metallopeptidase (MMP) activation [39–41]. MMPs may play a role in the pathogenesis of pulmonary function decline and previous research has shown significantly increased levels of MMPs in both serum and airways [42]. Proteolytic imbalance leads to emphysema development, small airway obstruction and increased sputum levels [43].

In our study, we found no statistically significant association between lung function reduction according to LLN and biomarkers previously associated with lung function decline. Interleukin 6 (IL6) is an acute phase response cytokine released from epithelial cells and macrophages, among other cells, and has in previous researched been positively associated with decreased lung function. Yasuda et al. found a significant increase of IL-6 in serum in both severe and mild/moderate COPD subjects compared to healthy age- and sex-matched controls, and Zeng et al. found a significant increase in stable COPD subjects compared to controls [44, 45]. As these studies compared subjects with diagnosed COPD with controls, increased inflammatory cytokines could be expected. Our study population is based on sample from overall healthy older subjects. Therefore, some participants with pulmonary function decline could be asymptomatic: only 60.3% of subjects fulfilling pulmonary function decline criteria reported being diagnosed with COPD. Thus, we could expect that the overall inflammatory level to be lower in our subjects compared to the above mentioned studies.

Tumor necrosis factor (TNF) is a pro-inflammatory cytokine secreted mainly by macrophages, monocytes, neutrophils, activated T-cells and NK-cells [46]. Increased serum levels of TNF have previously been associated with COPD in a male patient sample (n  =  30) with stable COPD and reduced body weight [46, 47]. The current study could not find a statistically significant association between serum TNF levels and airflow obstruction. Our study population was fit enough to visit the study centre, indicating a physical status without significant weight loss and where increased TNF levels would not be expected.

Surfactant protein D (SFTPD) is produced by pneumocytes and plays a role in the defence against microorganisms. Sin et al. studied 23 patients with advanced COPD (mean age 64.8 years, 12 females) and found an inverse association between FEV1 and serum levels of SFTPD. Ciftci et al. found significant increased serum levels of SFTPD in 63 stable COPD subjects (mean age 63.7 years, 5 females) compared to 25 controls (mean age 53.8 years, 8 females). Our study showed no significant association between serum levels of SFTPD and pulmonary function decline. Our study population was larger and had a more balanced gender distribution, which could partially explain that our results differ from previous research.

Our study has several strengths. The study population consisted of a general geriatric sample of subjects living in urban areas in southern Sweden, which allow us to investigate the association of pulmonary function decline and biomarkers in a representative sample. We defined pulmonary function decline according to LLN criteria as proposed by ERS [5]. The GOLD definition has a tendency to overestimate the prevalence of airflow obstruction in subjects aged  >  50 years [30]. A decreased pulmonary function with age is a physiological change and does not necessarily means that an elderly person has a disease. In addition, sex has been shown to affect LLN significantly, but this is not considered in the GOLD criteria [13]. In the current study, 54.7% were females. Despite the mentioned advantages of the LNN criteria, this definition somewhat underestimates the prevalence of COPD in the elderly [32]. We observed that 6.2% of subjects with FEV1/FVC  ≥  LLN had been diagnosed with COPD. Therefore, we believe that a potential underestimation of clinically diagnosed COPD in our study is negligible. Furthermore, a Belgian study of 567 individuals aged  ≥  80 years did not show a significantly increased risk of missing patients with increased risk of hospitalization or mortality when defining airflow limitation according to LLN instead of GOLD [48]. Similarly, a Finnish study could not show any clear difference in mortality risk between patients with airflow limitation according to GOLD criteria compared to patients with LLN-based airflow limitation definition [49]. We found the airflow obstruction prevalence according to LLN criteria to be 10.3% in our study, which is in accordance with COPD prevalence in the Swedish BOLD study, but higher than the prevalence in the study by Backman et al. [11]. The increased airflow obstruction prevalence could be explained by the fact that the current study only included subjects aged  >  65 years.

Our study has also some limitations. Incomplete data was due to mainly two factors: non-acceptable Olink QC and missing spirometry data. Olink QC was non-acceptable if the blood sample was too small or if the qPCR failed. These factors are not related to the subjects neither to their pulmonary function, and therefore are not expected to influence the presented results. Spirometry data was missing if the subject could not perform an acceptable manoeuvre which could indicate decreased pulmonary function due to the physical status. To minimize the exclusion of frail subjects, only one acceptable spirometry manoeuvre was required.

## Conclusion

In conclusion, our study showed a statistically significant association between elevated serum levels of PLAUR and decreased pulmonary function. Further research is needed to investigate the possible role of PLAUR in the pathogenesis in pulmonary function decline, as well as PLAUR as a potential marker for pulmonary decrease.

## Supplementary Information

**Additional file 1: Figure S1.** Pearson product-moment correlation coefficient across the proteins in the CVD II panel. The names of the proteins are listed below. **Figure S2.** Pearson product-moment correlation coefficient across the proteins in the CVD III panel. The names of the proteins are listed below. **Figure S3.** Pearson product-moment correlation coefficient across the proteins in the inflammation panel. The names of the proteins are listed below. **Table S1.** Results of the crude logistic model for all proteins. The number of observations is 611 in all models. **Table S2.** Results of the sensitivity analysis. The crude logistic model was calculated excluding all values below the lower limit of detection.

## Data Availability

Data availability and data use agreement policy follow approval from the GÅS committee (https://www.geriatrik.lu.se) and a relevant Ethics Committee in accordance to the General Data Protection Regulation rules.

## Abbreviations

COPD: Chronic obstructive pulmonary disease
CVD: Cardiovascular disease
WHO: World Health Organization
GOLD: Global Initiative for Chronic Obstructive Lung Disease
FEV1: Forced expiratory volume in 1 s
FVC: Forced vital capacity
ERS: European Respirator Society
LLN: Lower limit of normal
GÅS: Good ageing in Skåne (Gott åldrande i Skåne)
QC: Quality control
PEA: Proximity extension assay
ATS: American Thoracic Society
FDR: False positive discovery
PLAUR: Plasminogen activator, urokinase receptor
MMP: Matrix metallopeptidase
ECM: Extracellular matrix
TNF: Tumor necrosis factor
SFTPD: Surfactant protein D
TIA: Transient ischemic attack
BMI: Body mass index

## References

[1] WHO. Chronic obstructive pulmonary disease (COPD). 2017. https://www.who.int/news-room/fact-sheets/detail/chronic-obstructive-pulmonary-disease-(copd). Accessed 22 Dec 2020.

[2] Vogelmeier CF, Criner GJ, Martinez FJ, Anzueto A, Barnes PJ, Bourbeau J (2017). Global strategy for the diagnosis, management, and prevention of chronic obstructive lung disease 2017 report. GOLD executive summary. Am J Respir Crit Care Med.

[3] Thomas ET, Guppy M, Straus SE, Bell KJL, Glasziou P (2019). Rate of normal lung function decline in ageing adults: a systematic review of prospective cohort studies. BMJ Open.

[4] Quanjer PH, Stanojevic S, Cole TJ, Baur X, Hall GL, Culver BH (2012). Multi-ethnic reference values for spirometry for the 3–95-yr age range: the global lung function 2012 equations. Eur Respir J.

[5] Pellegrino R, Viegi G, Brusasco V, Crapo RO, Burgos F, Casaburi R (2005). Interpretative strategies for lung function tests. Eur Respir J.

[6] Bakke PS, Rönmark E, Eagan T, Pistelli F, Annesi-Maesano I, Maly M (2011). Recommendations for epidemiological studies on COPD. Eur Respir J.

[7] Viegi G, Pedreschi M, Pistelli F, Di Pede F, Baldacci S, Carrozzi L (2000). Prevalence of airways obstruction in a general population: European Respiratory Society vs American Thoracic Society definition. Chest.

[8] Schirnhofer L, Lamprecht B, Vollmer WM, Allison MJ, Studnicka M, Jensen RL (2007). COPD prevalence in Salzburg, Austria: results from the Burden of Obstructive Lung Disease (BOLD) Study. Chest.

[9] Vanfleteren LE, Franssen FM, Wesseling G, Wouters EF (2012). The prevalence of chronic obstructive pulmonary disease in Maastricht, the Netherlands. Respir Med.

[10] Danielsson P, Ólafsdóttir IS, Benediktsdóttir B, Gíslason T, Janson C (2012). The prevalence of chronic obstructive pulmonary disease in Uppsala, Sweden–the Burden of Obstructive Lung Disease (BOLD) study: cross-sectional population-based study. Clin Respir J.

[11] Backman H, Vanfleteren L, Lindberg A, Ekerljung L, Stridsman C, Axelsson M (2020). Decreased COPD prevalence in Sweden after decades of decrease in smoking. Respir Res.

[12] Lundbäck B, Lindberg A, Lindström M, Rönmark E, Jonsson AC, Jönsson E (2003). Not 15 but 50% of smokers develop COPD?–report from the obstructive lung disease in Northern Sweden Studies. Respir Med.

[13] Luoto JA, Elmståhl S, Wollmer P, Pihlsgård M (2015). Incidence of airflow limitation in subjects 65–100 years of age. Eur Respir J.

[14] Larsson K (2014). KOL: kroniskt obstruktiv lungsjukdom.

[15] Dickens JA, Miller BE, Edwards LD, Silverman EK, Lomas DA, Tal-Singer R (2011). COPD association and repeatability of blood biomarkers in the ECLIPSE cohort. Respir Res.

[16] Paone G, Leone V, Conti V, De Marchis L, Ialleni E, Graziani C, Salducci M (2016). Blood and sputum biomarkers in COPD and asthma: a review. Eur Rev Med Pharmacol Sci.

[17] MeSH. Karolinska Institutet. https://mesh.kib.ki.se/term/D007249/inflammation. Accessed 22 Dec 2020.

[18] Thompson AB, Daughton D, Robbins RA, Ghafouri MA, Oehlerking M, Rennard SI (1989). Intraluminal airway inflammation in chronic bronchitis. Characterization and correlation with clinical parameters. Am Rev Respir Dis.

[19] Cosio MG, Hale KA, Niewoehner DE (1980). Morphologic and morphometric effects of prolonged cigarette smoking on the small airways. Am Rev Respir Dis.

[20] Saetta M, Ghezzo H, Kim WD, King M, Angus GE, Wang NS (1985). Loss of alveolar attachments in smokers. A morphometric correlate of lung function impairment. Am Rev Respir Dis.

[21] Klein JS, Gamsu G, Webb WR, Golden JA, Müller NL (1992). High-resolution CT diagnosis of emphysema in symptomatic patients with normal chest radiographs and isolated low diffusing capacity. Radiology.

[22] Liu Y, Liu H, Li C, Ma C, Ge W (2020). Proteome profiling of lung tissues in chronic obstructive pulmonary disease (COPD): platelet and macrophage dysfunction contribute to the pathogenesis of COPD. Int J Chron Obstruct Pulmon Dis.

[23] Xing L, Xue Y, Yang Y, Wu P, Wong CCL, Wang H (2020). TMT-based quantitative proteomic analysis identification of integrin alpha 3 and integrin alpha 5 as novel biomarkers in pathogenesis of acute aortic dissection. Biomed Res Int.

[24] Ekström H, Elmståhl S (2006). Pain and fractures are independently related to lower walking speed and grip strength: results from the population study “Good Ageing in Skåne”. Acta Orthopedica.

[25] Assarsson E, Lundberg M, Holmquist G, Björkesten J, Bucht Thorsen S, Ekman D (2014). Homogenous 96-Plex PEA immunoassay exhibiting high sensitivity, specificity, and excellent scalability. PLoS ONE.

[26] Olink. Olink target 96 and target 48 panels for protein biomarker discovery. https://www.olink.com/products/target/. Accessed 20 Mar 2021.

[27] Olink. Proximity extension assay (PEA) technology. https://www.olink.com/data-you-can-trust/technology/. Accessed 22 Dec 2020.

[28] American Thoracic Society (1995). Standardization of spirometry, 1994 update. Am J Respir Crit Care Med.

[29] Fragoso CA (2016). Epidemiology of chronic obstructive pulmonary disease (COPD) in aging populations. COPD.

[30] Quanjer PH, Stanojevic S, Cole TJ, Baur X, Hall GL, Culver BH (2012). Multi-ethnic reference values for spirometry for the 3–95-yr age range: the global lung function 2012 equations. Eur Respir J.

[31] https://www.ers-education.org/guidelines/global-lung-function-initiative/spirometry-tools.aspx. Accessed 20 Nov 2020.

[32] Güder G, Brenner S, Angermann CE, Ertl G, Held M, Sachs AP (2012). GOLD or lower limit of normal definition? A comparison with expert-based diagnosis of chronic obstructive pulmonary disease in a prospective cohort-study. Respir Res.

[33] Benjamini Y, Yekutieli D (2001). The control of the false discovery rate in multiple testing under dependency. Ann Stat.

[34] Newson R (2010). Frequentist Q-values for multiple-test procedures. Stata J.

[35] Can Ü, Güzelant A, Yerlikaya FH, Yosunkaya Ş (2014). The role of serum soluble urokinase-type plasminogen activator receptor in stable chronic obstructive pulmonary disease. J Investig Med Off Publ Am Fed Clin Res.

[36] Xiao W, Hsu YP, Ishizaka A, Kirikae T, Moss RB (2005). Sputum cathelicidin, urokinase plasminogen activation system components, and cytokines discriminate cystic fibrosis, COPD, and asthma inflammation. Chest.

[37] Böcskei RM, Benczúr B, Losonczy G, Illyés M, Cziráki A, Müller V (2019). Soluble urokinase-type plasminogen activator receptor and arterial stiffness in patients with COPD. Lung.

[38] Loukeri A, Spithakis P-D, Moschos C, Loukeri P, Bartzeliotou A, Tzagkaraki A (2016). Plasma levels of soluble urokinase plasminogen activator receptor (suPAR) as a possible biomarker for lung cancer and/or COPD. Eur Respir J.

[39] Mondino A, Blasi F (2004). uPA and uPAR in fibrinolysis, immunity and pathology. Trends Immunol.

[40] Thunø M, Macho B, Eugen-Olsen J (2009). suPAR: the molecular crystal ball. Dis Markers.

[41] Stewart CE, Sayers I (2009). Characterisation of urokinase plasminogen activator receptor variants in human airway and peripheral cells. BMC Mol Biol.

[42] Linder R, Rönmark E, Pourazar J, Behndig A, Blomberg A, Lindberg A (2015). Serum metalloproteinase-9 is related to COPD severity and symptoms—cross-sectional data from a population based cohort-study. Respir Res.

[43] Kraen M, Frantz S, Nihlén U, Engström G, Löfdahl CG, Wollmer P (2019). Matrix metalloproteinases in COPD and atherosclerosis with emphasis on the effects of smoking. PLoS ONE.

[44] Yasuda N, Gotoh K, Minatoguchi S, Asano K, Nishigaki K, Nomura M (1998). An increase of soluble Fas, an inhibitor of apoptosis, associated with progression of COPD. Respir Med.

[45] Zeng YY, Hu WP, Zuo YH, Wang XR, Zhang J (2019). Altered serum levels of type I collagen turnover indicators accompanied by IL-6 and IL-8 release in stable COPD. Int J Chron Obstruct Pulmon Dis.

[46] Owen JA, Punt J, Stranford SA, Jones PP, Kuby J (2013). Kuby immunology.

[47] Di Francia M, Barbier D, Mege JL, Orehek J (1994). Tumor necrosis factor-alpha levels and weight loss in chronic obstructive pulmonary disease. Am J Respir Crit Care Med.

[48] Turkeshi E, Vaes B, Andreeva E, Matheï C, Adriaensen W, Van Pottelbergh G (2015). Airflow limitation by the Global Lungs Initiative equations in a cohort of very old adults. Eur Respir J.

[49] Mattila T, Vasankari T, Kanervisto M, Laitinen T, Impivaara O, Rissanen H (2015). Association between all-cause and cause-specific mortality and the GOLD stages 1–4: a 30-year follow-up among Finnish adults. Respir Med.

