# Supplementary material for: Association between cardiovascular disease- and inflammation-related serum biomarkers and poor lung function in elderly
Source: Clin Proteomics. 2021 Sep 28;18:23. doi: 10.1186/s12014-021-09329-7 (PMC8480099; doi:10.1186/s12014-021-09329-7)
Supplement: Supplementary file 1 — Additional file 1: Figure S1. Pearson product-moment correlation coefficient across the proteins in the CVD II panel. The names of the proteins are listed below. Figure S2. Pearson product-moment correlation coefficient across the proteins in the CVD III panel. The names of the proteins are listed below. Figure S3. Pearson product-moment correlation coefficient across the proteins in the inflammation panel. The names of the proteins are listed below. Table S1. Results of the crude logistic model for all proteins. The number of observations is 611 in all models. Table S2. Results of the sensitivity analysis. The crude logistic model was calculated excluding all values below the lower limit of detection. [file 12014_2021_9329_MOESM1_ESM.docx]

**Appendix**

Figure S1: Pearson product-moment correlation coefficient across the proteins in the CVD II panel. The names of the proteins are listed below.


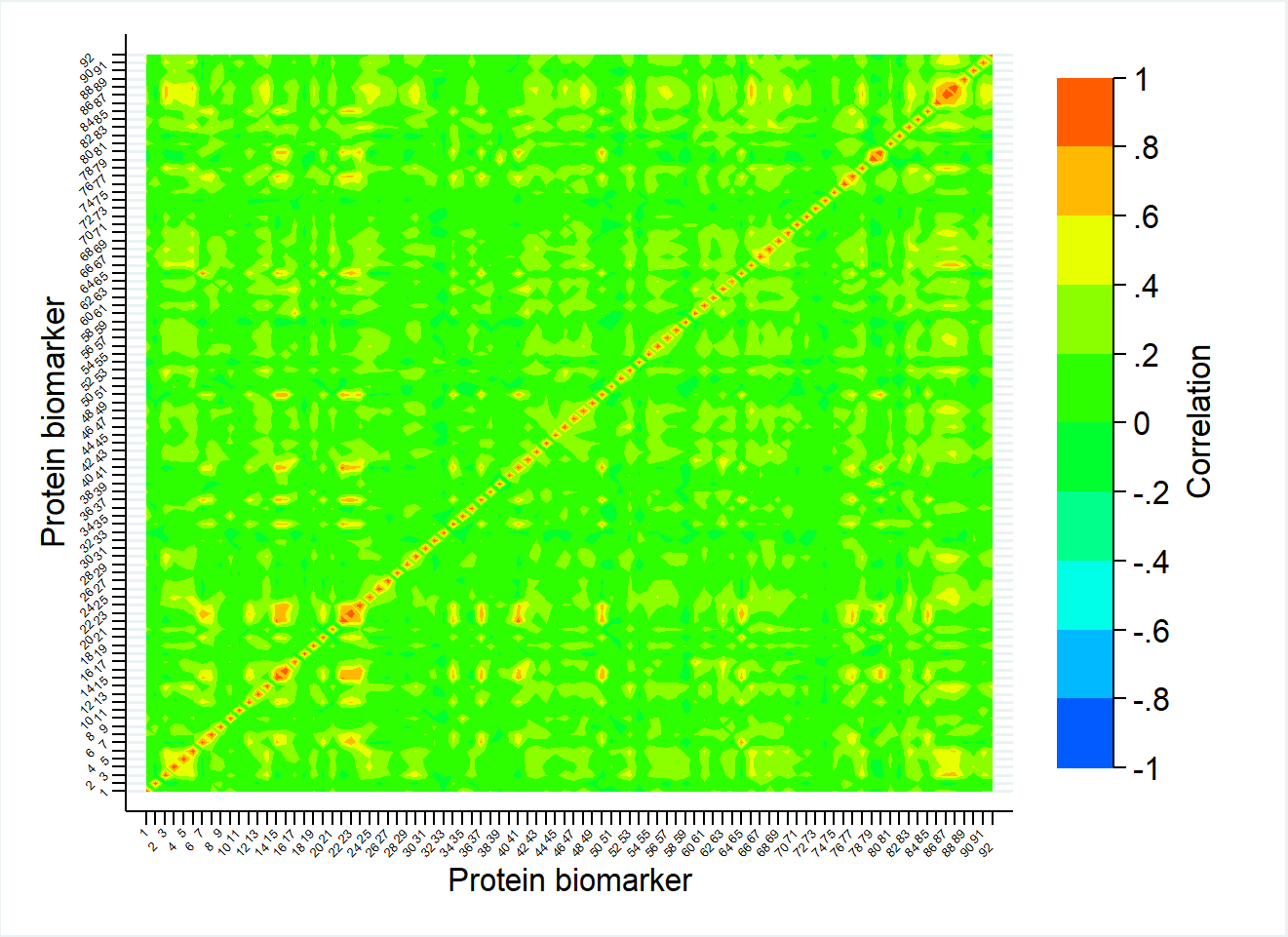


| Number | Protein name | Number | Protein name | Number | Protein name |
| --- | --- | --- | --- | --- | --- |
| 1 | ACE2 | 31 | FST | 61 | OSCAR |
| 2 | ADAMTS13 | 32 | GDF2 | 62 | PAPPA |
| 3 | ADM | 33 | GH1 | 63 | PARP1 |
| 4 | AGER | 34 | GLO1 | 64 | PDCD1LG2 |
| 5 | AGRP | 35 | HAO1 | 65 | PDGFB |
| 6 | AMBP | 36 | HAVCR1 | 66 | PGF |
| 7 | ANGPT1 | 37 | HBEGF | 67 | PIGR |
| 8 | BMP6 | 38 | HMOX1 | 68 | PRELP |
| 9 | BOC | 39 | HSPB1 | 69 | PRSS27 |
| 10 | CA5A | 40 | IDUA | 70 | PRSS8 |
| 11 | CBLIF | 41 | IKBKG | 71 | PTX3 |
| 12 | CCL17 | 42 | IL16 | 72 | REN |
| 13 | CCL3 | 43 | IL17D | 73 | SELPLG |
| 14 | CD4 | 44 | IL18 | 74 | SERPINA12 |
| 15 | CD40LG | 45 | IL1RL2 | 75 | SLAMF7 |
| 16 | CD84 | 46 | IL1RN | 76 | SOD2 |
| 17 | CEACAM8 | 47 | IL27 | 77 | SORT1 |
| 18 | CTRC | 48 | IL4R | 78 | SPON2 |
| 19 | CTSL | 49 | IL6 | 79 | SRC |
| 20 | CXCL1 | 50 | ITGB1BP2 | 80 | STK4 |
| 21 | DCN | 51 | KITLG | 81 | TEK |
| 22 | DECR1 | 52 | LEP | 82 | TGM2 |
| 23 | DKK1 | 53 | LGALS9 | 83 | THBD |
| 24 | F2R | 54 | LPL | 84 | THBS2 |
| 25 | F3 | 55 | MARCO | 85 | THPO |
| 26 | FABP2 | 56 | MERTK | 86 | TNFRSF10A |
| 27 | FABP6 | 57 | MMP12 | 87 | TNFRSF10B |
| 28 | FCGR2B | 58 | MMP7 | 88 | TNFRSF11A |
| 29 | FGF21 | 59 | NPPB | 89 | TNFRSF13B |
| 30 | FGF23 | 60 | OLR1 | 90 | VEGFD |
|  |  |  |  | 91 | VSIG2 |
|  |  |  |  | 92 | XCL1 |

Figure S2: Pearson product-moment correlation coefficient across the proteins in the CVD III panel. The names of the proteins are listed below.


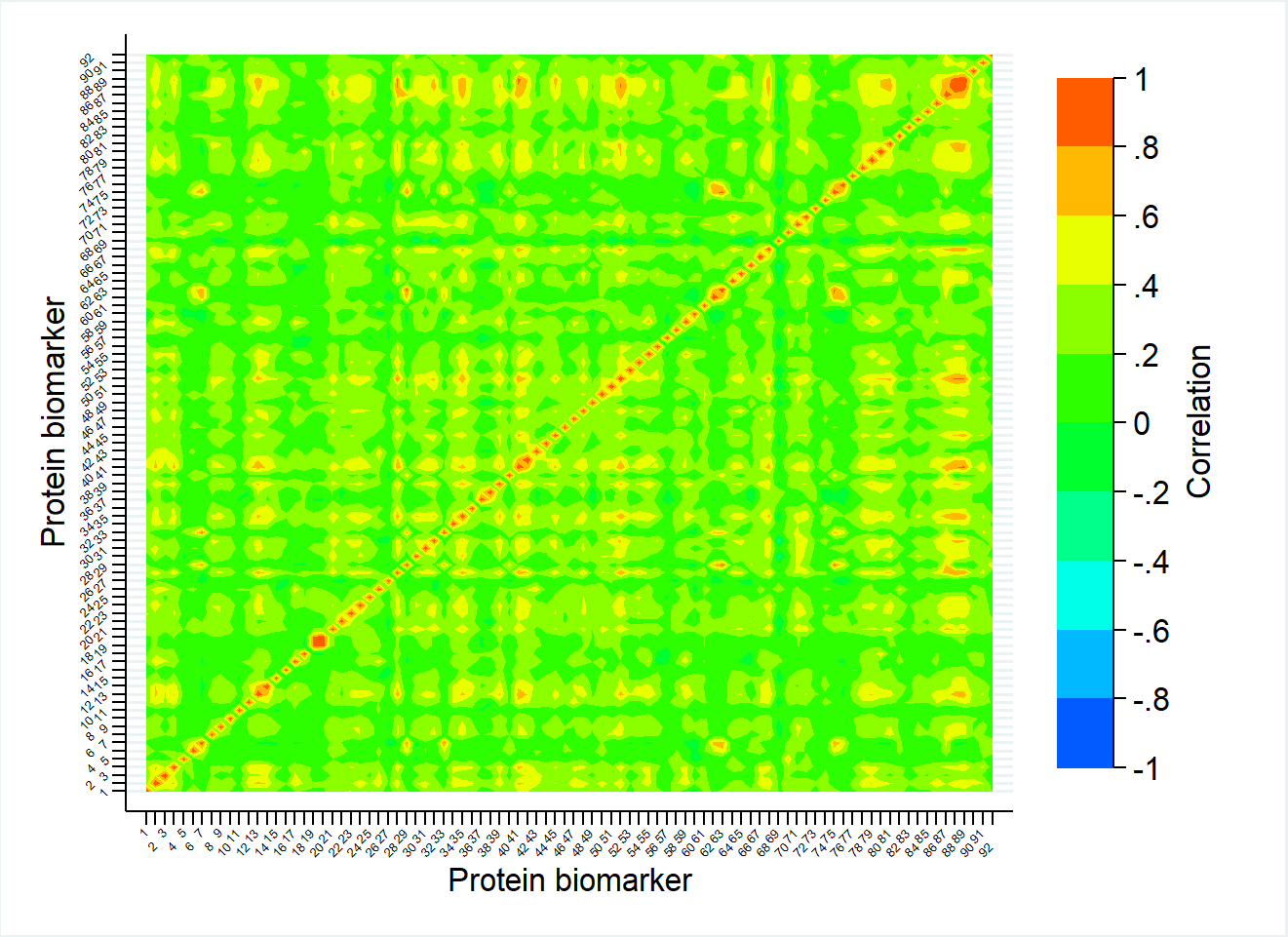


| Number | Protein name | Number | Protein name | Number | Protein name |
| --- | --- | --- | --- | --- | --- |
| 1 | ACP5 | 31 | FAS | 61 | PCSK9 |
| 2 | ALCAM | 32 | GDF15 | 62 | PDGFA |
| 3 | ANPEP | 33 | GP6 | 63 | PECAM1 |
| 4 | AXL | 34 | GRN | 64 | PGLYRP1 |
| 5 | AZU1 | 35 | HSPG2 | 65 | PI3 |
| 6 | BLMH | 36 | ICAM2 | 66 | PLAT |
| 7 | CASP3 | 37 | IGFBP1 | 67 | PLAU |
| 8 | CCL15 | 38 | IGFBP2 | 68 | PLAUR |
| 9 | CCL16 | 39 | IGFBP7 | 69 | PON3 |
| 10 | CCL2 | 40 | IL17RA | 70 | PRTN3 |
| 11 | CCL24 | 41 | IL18BP | 71 | RARRES2 |
| 12 | CD163 | 42 | IL1R1 | 72 | RETN |
| 13 | CD93 | 43 | IL1R2 | 73 | SCGB3A2 |
| 14 | CDH5 | 44 | IL1RL1 | 74 | SELE |
| 15 | CHI3L1 | 45 | IL2RA | 75 | SELP |
| 16 | CHIT1 | 46 | IL6R | 76 | SERPINE1 |
| 17 | CNTN1 | 47 | ITGB2 | 77 | SFTPD |
| 18 | COL1A1 | 48 | KLK6 | 78 | SIRPA |
| 19 | CPA1 | 49 | LDLR | 79 | SPON1 |
| 20 | CPB1 | 50 | LGALS3 | 80 | SPP1 |
| 21 | CSTB | 51 | LGALS4 | 81 | TFF3 |
| 22 | CTSD | 52 | LTBR | 82 | TFPI |
| 23 | CTSZ | 53 | MB | 83 | TFRC |
| 24 | CXCL16 | 54 | MEPE | 84 | TIMP4 |
| 25 | DLK1 | 55 | MMP2 | 85 | TNFRSF10C |
| 26 | EGFR | 56 | MMP3 | 86 | TNFRSF11B |
| 27 | EPCAM | 57 | MMP9 | 87 | TNFRSF14 |
| 28 | EPHB4 | 58 | MPO | 88 | TNFRSF1A |
| 29 | F11R | 59 | NOTCH3 | 89 | TNFRSF1B |
| 30 | FABP4 | 60 | NPPB | 90 | TNFSF13B |
|  |  |  |  | 91 | TREML2 |
|  |  |  |  | 92 | VWF |

Figure S3: Pearson product-moment correlation coefficient across the proteins in the inflammation panel. The names of the proteins are listed below.


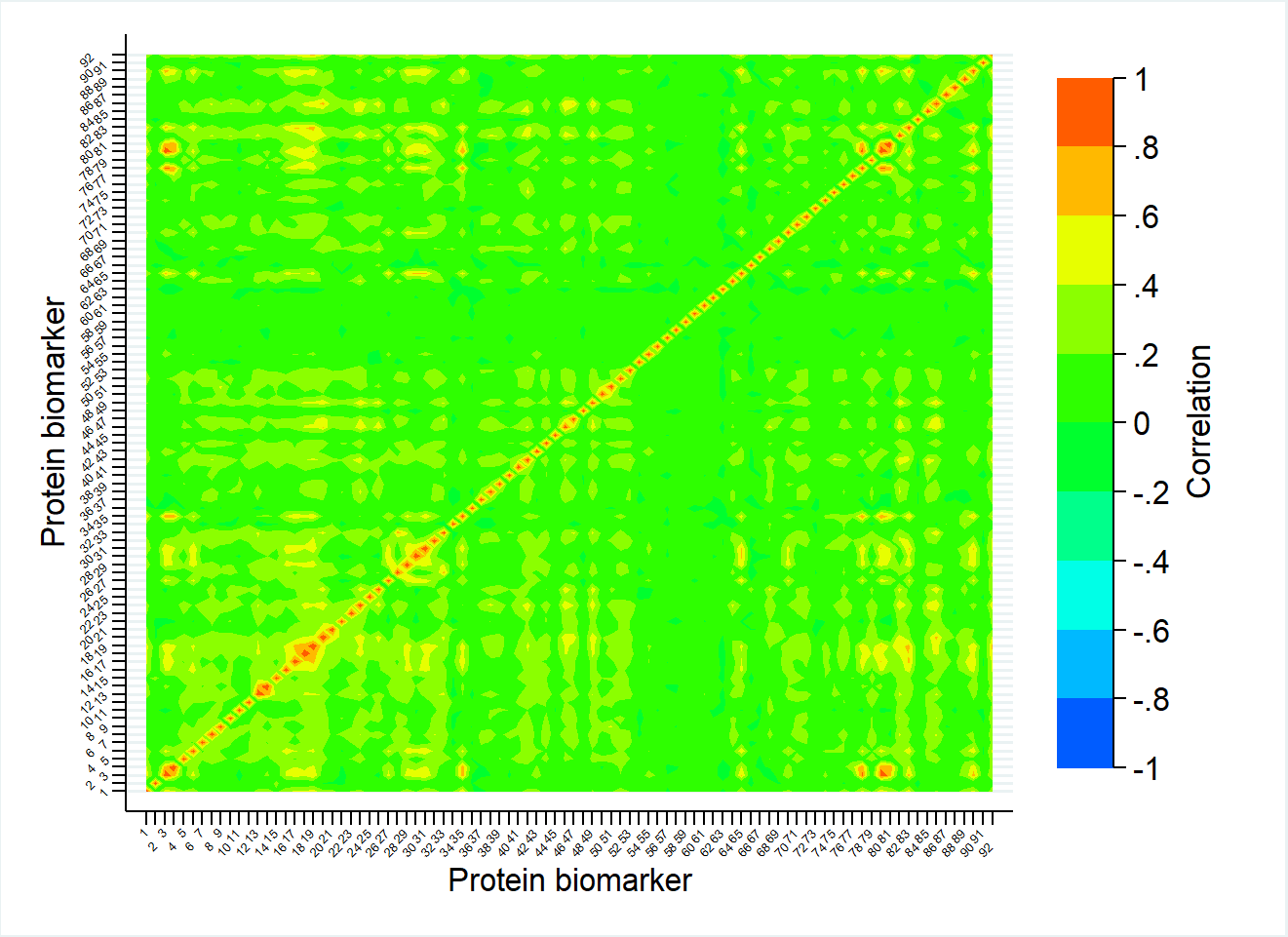


| Number | Protein name | Number | Protein name | Number | Protein name |
| --- | --- | --- | --- | --- | --- |
| 1 | ADA | 31 | CXCL6 | 61 | IL33 |
| 2 | ARTN | 32 | CXCL8 | 62 | IL4 |
| 3 | AXIN1 | 33 | CXCL9 | 63 | IL5 |
| 4 | CASP8 | 34 | DNER | 64 | IL6 |
| 5 | CCL11 | 35 | EIF4EBP1 | 65 | IL7 |
| 6 | CCL13 | 36 | FGF19 | 66 | KITLG |
| 7 | CCL19 | 37 | FGF21 | 67 | LIF |
| 8 | CCL2 | 38 | FGF23 | 68 | LIFR |
| 9 | CCL20 | 39 | FGF5 | 69 | LTA |
| 10 | CCL23 | 40 | FLT3LG | 70 | MMP1 |
| 11 | CCL25 | 41 | GDNF | 71 | MMP10 |
| 12 | CCL28 | 42 | HGF | 72 | NGF |
| 13 | CCL3 | 43 | IFNG | 73 | NRTN |
| 14 | CCL4 | 44 | IL10 | 74 | NTF3 |
| 15 | CCL7 | 45 | IL10RA | 75 | OSM |
| 16 | CCL8 | 46 | IL10RB | 76 | PLAU |
| 17 | CD244 | 47 | IL12B | 77 | S100A12 |
| 18 | CD274 | 48 | IL13 | 78 | SIRT2 |
| 19 | CD40 | 49 | IL15RA | 79 | SLAMF1 |
| 20 | CD5 | 50 | IL17A | 80 | STAMBP |
| 21 | CD6 | 51 | IL17C | 81 | SULT1A1 |
| 22 | CD8A | 52 | IL18 | 82 | TGFA |
| 23 | CDCP1 | 53 | IL18R1 | 83 | TGFB1 |
| 24 | CSF1 | 54 | IL1A | 84 | TNF |
| 25 | CST5 | 55 | IL2 | 85 | TNFRSF11B |
| 26 | CX3CL1 | 56 | IL20 | 86 | TNFRSF9 |
| 27 | CXCL1 | 57 | IL20RA | 87 | TNFSF10 |
| 28 | CXCL10 | 58 | IL22RA1 | 88 | TNFSF11 |
| 29 | CXCL11 | 59 | IL24 | 89 | TNFSF12 |
| 30 | CXCL5 | 60 | IL2RB | 90 | TNFSF14 |
|  |  |  |  | 91 | TSLP |
|  |  |  |  | 92 | VEGFA |

**Table S1**: Results of the crude logistic model for all proteins. The number of observations is 611 in all models.

| Panel | Protein | Model coefficient | Odds ratio (Ref: FEV1/FVC> LLN) | raw p-value | P-value adjusted by Yekutieli method |
| --- | --- | --- | --- | --- | --- |
|  |  |  |  |  |  |
| CVD II | PTX3 | 1,1073 | 3,0262 | 0,0008 | 0,3860 |
|  | TNFRSF10A | 0,8393 | 2,3147 | 0,0063 | 1,0000 |
|  | VEGFD | 1,1146 | 3,0482 | 0,0100 | 1,0000 |
|  | LPL | 0,9002 | 2,4601 | 0,0115 | 1,0000 |
|  | FGF23 | 0,4361 | 1,5466 | 0,0120 | 1,0000 |
|  | TNFRSF10B | 0,6791 | 1,9721 | 0,0130 | 1,0000 |
|  | VSIG2 | 0,5086 | 1,6630 | 0,0162 | 1,0000 |
|  | XCL1 | 0,5532 | 1,7388 | 0,0188 | 1,0000 |
|  | CTRC | -0,3516 | 0,7035 | 0,0196 | 1,0000 |
|  | CCL17 | 0,3162 | 1,3719 | 0,0226 | 1,0000 |
|  | MMP12 | 0,4040 | 1,4978 | 0,0247 | 1,0000 |
|  | KITLG | -0,8418 | 0,4309 | 0,0276 | 1,0000 |
|  | IL27 | 0,8113 | 2,2508 | 0,0310 | 1,0000 |
|  | OLR1 | 0,4636 | 1,5898 | 0,0341 | 1,0000 |
|  | GH1 | 0,1628 | 1,1768 | 0,0366 | 1,0000 |
|  | OSCAR | 1,2689 | 3,5569 | 0,0388 | 1,0000 |
|  | MMP7 | 0,6499 | 1,9153 | 0,0447 | 1,0000 |
|  | ANGPT1 | 0,3017 | 1,3522 | 0,0467 | 1,0000 |
|  | FGF21 | 0,1969 | 1,2176 | 0,0500 | 1,0000 |
|  | HBEGF | 0,3801 | 1,4624 | 0,0512 | 1,0000 |
|  | IDUA | 0,5865 | 1,7976 | 0,0513 | 1,0000 |
|  | IL17D | 0,7984 | 2,2219 | 0,0535 | 1,0000 |
|  | PDGFB | 0,3135 | 1,3682 | 0,0662 | 1,0000 |
|  | SORT1 | 0,7634 | 2,1456 | 0,0821 | 1,0000 |
|  | THPO | 0,5360 | 1,7091 | 0,0874 | 1,0000 |
|  | FABP6 | 0,2831 | 1,3272 | 0,0898 | 1,0000 |
|  | LEP | -0,2031 | 0,8162 | 0,0930 | 1,0000 |
|  | BOC | 0,8945 | 2,4460 | 0,0954 | 1,0000 |
|  | MERTK | -0,6605 | 0,5166 | 0,0971 | 1,0000 |
|  | SPON2 | 1,2607 | 3,5277 | 0,0985 | 1,0000 |
|  | DKK1 | 0,3360 | 1,3994 | 0,1071 | 1,0000 |
|  | GLO1 | 0,2720 | 1,3126 | 0,1184 | 1,0000 |
|  | F3 | 0,6650 | 1,9444 | 0,1207 | 1,0000 |
|  | SLAMF7 | 0,2899 | 1,3364 | 0,1515 | 1,0000 |
|  | AGRP | 0,4925 | 1,6364 | 0,1734 | 1,0000 |
|  | CTSL | 0,5556 | 1,7430 | 0,1749 | 1,0000 |
|  | THBS2 | 0,7622 | 2,1430 | 0,2060 | 1,0000 |
|  | CEACAM8 | 0,2572 | 1,2933 | 0,2117 | 1,0000 |
|  | FABP2 | 0,2035 | 1,2257 | 0,2120 | 1,0000 |
|  | CD84 | 0,4033 | 1,4968 | 0,2170 | 1,0000 |
|  | TNFRSF13B | 0,3373 | 1,4011 | 0,2274 | 1,0000 |
|  | DCN | -0,5655 | 0,5681 | 0,2322 | 1,0000 |
|  | ITGB1BP2 | 0,1310 | 1,1399 | 0,2386 | 1,0000 |
|  | TNFRSF11A | 0,2933 | 1,3408 | 0,2406 | 1,0000 |
|  | IL6 | 0,1739 | 1,1900 | 0,2450 | 1,0000 |
|  | CD40LG | 0,1356 | 1,1452 | 0,2603 | 1,0000 |
|  | HAO1 | -0,1253 | 0,8822 | 0,2614 | 1,0000 |
|  | PAPPA | 0,2574 | 1,2936 | 0,2841 | 1,0000 |
|  | IKBKG | 0,1229 | 1,1308 | 0,3051 | 1,0000 |
|  | HAVCR1 | 0,1740 | 1,1901 | 0,3062 | 1,0000 |
|  | DECR1 | 0,1001 | 1,1053 | 0,3114 | 1,0000 |
|  | CCL3 | 0,1999 | 1,2212 | 0,3153 | 1,0000 |
|  | NPPB | 0,0833 | 1,0869 | 0,3201 | 1,0000 |
|  | CXCL1 | 0,1917 | 1,2113 | 0,3220 | 1,0000 |
|  | FCGR2B | 0,1651 | 1,1795 | 0,3540 | 1,0000 |
|  | PRSS8 | -0,2974 | 0,7428 | 0,3900 | 1,0000 |
|  | PRELP | 0,5574 | 1,7461 | 0,4121 | 1,0000 |
|  | HSPB1 | -0,3087 | 0,7344 | 0,4206 | 1,0000 |
|  | IL1RN | -0,2096 | 0,8109 | 0,4219 | 1,0000 |
|  | ADAMTS13 | 0,7294 | 2,0737 | 0,4321 | 1,0000 |
|  | ADM | 0,2344 | 1,2642 | 0,4601 | 1,0000 |
|  | IL4R | 0,2817 | 1,3254 | 0,4638 | 1,0000 |
|  | PIGR | 0,6918 | 1,9973 | 0,4640 | 1,0000 |
|  | PARP1 | 0,1415 | 1,1520 | 0,4781 | 1,0000 |
|  | IL16 | 0,1926 | 1,2124 | 0,4840 | 1,0000 |
|  | LGALS9 | 0,2770 | 1,3192 | 0,5065 | 1,0000 |
|  | THBD | 0,2687 | 1,3083 | 0,5123 | 1,0000 |
|  | SRC | -0,1457 | 0,8644 | 0,5270 | 1,0000 |
|  | MARCO | 0,3758 | 1,4561 | 0,5615 | 1,0000 |
|  | AGER | 0,1718 | 1,1874 | 0,5664 | 1,0000 |
|  | GDF2 | 0,2214 | 1,2478 | 0,5695 | 1,0000 |
|  | SERPINA12 | -0,0698 | 0,9326 | 0,5877 | 1,0000 |
|  | AMBP | 0,3809 | 1,4636 | 0,5973 | 1,0000 |
|  | CBLIF | -0,0562 | 0,9454 | 0,6456 | 1,0000 |
|  | REN | 0,0640 | 1,0660 | 0,6586 | 1,0000 |
|  | SELPLG | 0,2144 | 1,2391 | 0,6712 | 1,0000 |
|  | IL18 | 0,1003 | 1,1056 | 0,6788 | 1,0000 |
|  | STK4 | 0,0534 | 1,0548 | 0,7252 | 1,0000 |
|  | PRSS27 | 0,1066 | 1,1125 | 0,7305 | 1,0000 |
|  | BMP6 | 0,0890 | 1,0930 | 0,7589 | 1,0000 |
|  | PGF | 0,1134 | 1,1200 | 0,7616 | 1,0000 |
|  | CD4 | 0,1114 | 1,1179 | 0,7912 | 1,0000 |
|  | HMOX1 | -0,0926 | 0,9116 | 0,8027 | 1,0000 |
|  | IL1RL2 | 0,0742 | 1,0771 | 0,8305 | 1,0000 |
|  | TGM2 | 0,0528 | 1,0542 | 0,8376 | 1,0000 |
|  | PDCD1LG2 | 0,0611 | 1,0630 | 0,8881 | 1,0000 |
|  | SOD2 | -0,1479 | 0,8625 | 0,9011 | 1,0000 |
|  | CA5A | -0,0137 | 0,9864 | 0,9404 | 1,0000 |
|  | ACE2 | -0,0073 | 0,9927 | 0,9755 | 1,0000 |
|  | F2R | -0,0101 | 0,9900 | 0,9804 | 1,0000 |
|  | TEK | 0,0110 | 1,0110 | 0,9852 | 1,0000 |
|  | FST | -0,0043 | 0,9957 | 0,9897 | 1,0000 |
|  |  |  |  |  |  |
| CVD III | PLAUR | 1,4756 | 4,3738 | 0,0000 | 0,0165 |
|  | SCGB3A2 | 0,6116 | 1,8434 | 0,0007 | 0,1569 |
|  | TNFSF13B | 1,1187 | 3,0609 | 0,0012 | 0,1822 |
|  | TFF3 | 0,7354 | 2,0863 | 0,0017 | 0,1822 |
|  | IGFBP2 | 0,7619 | 2,1423 | 0,0019 | 0,1822 |
|  | IL18BP | 1,1169 | 3,0553 | 0,0029 | 0,2301 |
|  | IGFBP1 | 0,3686 | 1,4457 | 0,0059 | 0,3814 |
|  | LGALS4 | 0,6163 | 1,8521 | 0,0077 | 0,3814 |
|  | SFTPD | 0,4736 | 1,6058 | 0,0085 | 0,3814 |
|  | SPP1 | 0,6978 | 2,0093 | 0,0088 | 0,3814 |
|  | COL1A1 | 0,9797 | 2,6637 | 0,0091 | 0,3814 |
|  | F11R | 0,2691 | 1,3087 | 0,0098 | 0,3814 |
|  | IL2RA | 0,7528 | 2,1229 | 0,0113 | 0,3814 |
|  | TNFRSF14 | 0,5855 | 1,7959 | 0,0114 | 0,3814 |
|  | GDF15 | 0,5257 | 1,6916 | 0,0128 | 0,3872 |
|  | GP6 | 0,5632 | 1,7564 | 0,0132 | 0,3872 |
|  | PECAM1 | 0,3600 | 1,4333 | 0,0155 | 0,4276 |
|  | PDGFA | 0,3883 | 1,4745 | 0,0180 | 0,4700 |
|  | SELP | 0,3688 | 1,4459 | 0,0225 | 0,5565 |
|  | TNFRSF1B | 0,6006 | 1,8232 | 0,0283 | 0,6428 |
|  | CCL15 | 0,5012 | 1,6507 | 0,0287 | 0,6428 |
|  | CHIT1 | 0,2917 | 1,3388 | 0,0307 | 0,6484 |
|  | TNFRSF11B | 0,9084 | 2,4802 | 0,0326 | 0,6484 |
|  | TNFRSF10C | 0,5951 | 1,8132 | 0,0331 | 0,6484 |
|  | MMP9 | 0,4052 | 1,4996 | 0,0362 | 0,6801 |
|  | IL1R1 | 0,9245 | 2,5207 | 0,0414 | 0,7472 |
|  | CXCL16 | 0,8136 | 2,2560 | 0,0470 | 0,7947 |
|  | PGLYRP1 | 0,5667 | 1,7624 | 0,0474 | 0,7947 |
|  | TIMP4 | 0,5814 | 1,7885 | 0,0514 | 0,8324 |
|  | KLK6 | 0,6400 | 1,8964 | 0,0564 | 0,8828 |
|  | ALCAM | 0,9409 | 2,5623 | 0,0722 | 1,0000 |
|  | TFPI | 0,7395 | 2,0950 | 0,0788 | 1,0000 |
|  | SPON1 | 0,7904 | 2,2044 | 0,0849 | 1,0000 |
|  | IGFBP7 | 0,6056 | 1,8323 | 0,0856 | 1,0000 |
|  | PRTN3 | 0,3990 | 1,4903 | 0,0936 | 1,0000 |
|  | TNFRSF1A | 0,4466 | 1,5630 | 0,0956 | 1,0000 |
|  | AXL | 0,6415 | 1,8993 | 0,1106 | 1,0000 |
|  | CTSZ | 0,5287 | 1,6967 | 0,1177 | 1,0000 |
|  | TREML2 | 0,4322 | 1,5406 | 0,1293 | 1,0000 |
|  | SERPINE1 | 0,2463 | 1,2792 | 0,1398 | 1,0000 |
|  | VWF | 0,2071 | 1,2301 | 0,1449 | 1,0000 |
|  | LTBR | 0,4872 | 1,6277 | 0,1566 | 1,0000 |
|  | CD93 | 0,6010 | 1,8239 | 0,1568 | 1,0000 |
|  | PLAU | 0,4322 | 1,5407 | 0,1671 | 1,0000 |
|  | CPA1 | -0,2399 | 0,7867 | 0,1698 | 1,0000 |
|  | CPB1 | -0,2368 | 0,7892 | 0,1812 | 1,0000 |
|  | CHI3L1 | 0,1833 | 1,2012 | 0,1832 | 1,0000 |
|  | CASP3 | 0,1733 | 1,1892 | 0,1838 | 1,0000 |
|  | MPO | 0,4585 | 1,5817 | 0,1842 | 1,0000 |
|  | RARRES2 | 0,6691 | 1,9524 | 0,1960 | 1,0000 |
|  | CTSD | 0,3155 | 1,3710 | 0,2103 | 1,0000 |
|  | IL17RA | 0,3135 | 1,3683 | 0,2138 | 1,0000 |
|  | ICAM2 | 0,4492 | 1,5671 | 0,2183 | 1,0000 |
|  | MMP2 | 0,4735 | 1,6055 | 0,2274 | 1,0000 |
|  | PCSK9 | 0,4697 | 1,5994 | 0,2301 | 1,0000 |
|  | MMP3 | 0,2246 | 1,2519 | 0,2598 | 1,0000 |
|  | EPHB4 | 0,4256 | 1,5305 | 0,2624 | 1,0000 |
|  | CSTB | 0,2467 | 1,2798 | 0,2695 | 1,0000 |
|  | CCL24 | 0,1641 | 1,1784 | 0,2754 | 1,0000 |
|  | CCL16 | 0,2788 | 1,3215 | 0,2760 | 1,0000 |
|  | FAS | 0,2737 | 1,3149 | 0,3100 | 1,0000 |
|  | ACP5 | 0,3512 | 1,4207 | 0,3216 | 1,0000 |
|  | LGALS3 | 0,3297 | 1,3906 | 0,3676 | 1,0000 |
|  | SIRPA | 0,2623 | 1,2999 | 0,4177 | 1,0000 |
|  | NOTCH3 | 0,2983 | 1,3476 | 0,4224 | 1,0000 |
|  | IL6R | 0,2788 | 1,3215 | 0,4299 | 1,0000 |
|  | IL1RL1 | 0,1826 | 1,2003 | 0,4615 | 1,0000 |
|  | HSPG2 | 0,3096 | 1,3629 | 0,4795 | 1,0000 |
|  | FABP4 | 0,1128 | 1,1194 | 0,5122 | 1,0000 |
|  | ITGB2 | -0,2531 | 0,7764 | 0,5197 | 1,0000 |
|  | CD163 | 0,1905 | 1,2098 | 0,5241 | 1,0000 |
|  | EGFR | -0,3493 | 0,7052 | 0,5754 | 1,0000 |
|  | DLK1 | 0,1151 | 1,1220 | 0,5962 | 1,0000 |
|  | PON3 | 0,1147 | 1,1215 | 0,6026 | 1,0000 |
|  | LDLR | -0,1266 | 0,8811 | 0,6178 | 1,0000 |
|  | EPCAM | 0,0544 | 1,0559 | 0,6602 | 1,0000 |
|  | RETN | 0,1180 | 1,1253 | 0,6711 | 1,0000 |
|  | TFRC | -0,1030 | 0,9021 | 0,6990 | 1,0000 |
|  | ANPEP | -0,1778 | 0,8371 | 0,7034 | 1,0000 |
|  | CNTN1 | -0,1492 | 0,8614 | 0,7069 | 1,0000 |
|  | BLMH | 0,0892 | 1,0933 | 0,7328 | 1,0000 |
|  | NPPB | 0,0362 | 1,0369 | 0,7432 | 1,0000 |
|  | CCL2 | 0,0731 | 1,0759 | 0,7516 | 1,0000 |
|  | MB | 0,0632 | 1,0652 | 0,7559 | 1,0000 |
|  | PI3 | -0,0446 | 0,9564 | 0,8203 | 1,0000 |
|  | PLAT | 0,0480 | 1,0491 | 0,8241 | 1,0000 |
|  | CDH5 | -0,0724 | 0,9302 | 0,8614 | 1,0000 |
|  | AZU1 | -0,0229 | 0,9774 | 0,8723 | 1,0000 |
|  | MEPE | 0,0301 | 1,0305 | 0,9186 | 1,0000 |
|  | IL1R2 | 0,0291 | 1,0296 | 0,9435 | 1,0000 |
|  | GRN | 0,0309 | 1,0314 | 0,9471 | 1,0000 |
|  | SELE | -0,0112 | 0,9889 | 0,9607 | 1,0000 |
|  |  |  |  |  |  |
| Inflammation | MMP1 | 0,4375 | 1,5489 | 0,0009 | 0,2712 |
|  | CXCL9 | 0,4647 | 1,5915 | 0,0014 | 0,2712 |
|  | CXCL11 | 0,4783 | 1,6134 | 0,0017 | 0,2712 |
|  | VEGFA | 0,9260 | 2,5244 | 0,0029 | 0,3416 |
|  | IL15RA | 1,3554 | 3,8783 | 0,0042 | 0,3989 |
|  | CCL13 | 0,5833 | 1,7920 | 0,0071 | 0,5553 |
|  | FGF23 | 0,4414 | 1,5549 | 0,0084 | 0,5633 |
|  | OSM | 0,4694 | 1,5991 | 0,0099 | 0,5800 |
|  | TNFRSF9 | 0,6194 | 1,8578 | 0,0112 | 0,5830 |
|  | IL17A | 0,3340 | 1,3966 | 0,0178 | 0,7002 |
|  | MMP10 | 0,5261 | 1,6924 | 0,0188 | 0,7002 |
|  | TGFA | 0,9247 | 2,5212 | 0,0203 | 0,7002 |
|  | CCL25 | 0,6046 | 1,8305 | 0,0208 | 0,7002 |
|  | TGFB1 | 0,6182 | 1,8555 | 0,0230 | 0,7002 |
|  | CCL11 | 0,7431 | 2,1025 | 0,0230 | 0,7002 |
|  | TNFSF14 | 0,4871 | 1,6277 | 0,0241 | 0,7002 |
|  | CXCL6 | 0,3770 | 1,4579 | 0,0253 | 0,7002 |
|  | IL24 | 0,2523 | 1,2869 | 0,0325 | 0,8486 |
|  | S100A12 | 0,3113 | 1,3652 | 0,0403 | 0,9777 |
|  | CD244 | 0,5680 | 1,7648 | 0,0416 | 0,9777 |
|  | KITLG | -0,7867 | 0,4553 | 0,0503 | 1,0000 |
|  | CXCL10 | 0,2838 | 1,3281 | 0,0508 | 1,0000 |
|  | CD5 | 0,7084 | 2,0307 | 0,0539 | 1,0000 |
|  | FGF21 | 0,2086 | 1,2319 | 0,0563 | 1,0000 |
|  | CCL20 | 0,2401 | 1,2714 | 0,0595 | 1,0000 |
|  | CSF1 | 1,2471 | 3,4801 | 0,0612 | 1,0000 |
|  | STAMBP | 0,2322 | 1,2614 | 0,0632 | 1,0000 |
|  | IL17C | 0,3037 | 1,3549 | 0,0675 | 1,0000 |
|  | NTF3 | 0,4568 | 1,5790 | 0,0728 | 1,0000 |
|  | GDNF | 0,5377 | 1,7121 | 0,0746 | 1,0000 |
|  | TNFRSF11B | 0,7635 | 2,1458 | 0,0754 | 1,0000 |
|  | CX3CL1 | 0,5663 | 1,7618 | 0,0773 | 1,0000 |
|  | AXIN1 | 0,1859 | 1,2043 | 0,0784 | 1,0000 |
|  | IL7 | 0,3543 | 1,4251 | 0,0820 | 1,0000 |
|  | CCL8 | 0,3811 | 1,4638 | 0,0970 | 1,0000 |
|  | IL10RB | 0,7458 | 2,1082 | 0,0992 | 1,0000 |
|  | CD40 | 0,4271 | 1,5328 | 0,1041 | 1,0000 |
|  | CCL3 | 0,3028 | 1,3536 | 0,1083 | 1,0000 |
|  | CCL28 | 0,6405 | 1,8975 | 0,1134 | 1,0000 |
|  | CXCL8 | 0,2657 | 1,3043 | 0,1360 | 1,0000 |
|  | NRTN | 0,3191 | 1,3758 | 0,1406 | 1,0000 |
|  | FGF5 | 0,7760 | 2,1728 | 0,1490 | 1,0000 |
|  | CCL2 | 0,4458 | 1,5617 | 0,1536 | 1,0000 |
|  | SIRT2 | 0,1605 | 1,1741 | 0,1561 | 1,0000 |
|  | IL10RA | 0,1941 | 1,2142 | 0,1562 | 1,0000 |
|  | IL10 | 0,3469 | 1,4146 | 0,1700 | 1,0000 |
|  | HGF | 0,4903 | 1,6329 | 0,1761 | 1,0000 |
|  | PLAU | 0,5594 | 1,7497 | 0,1901 | 1,0000 |
|  | TNFSF10 | 0,6363 | 1,8894 | 0,1906 | 1,0000 |
|  | CD274 | 0,3281 | 1,3884 | 0,2309 | 1,0000 |
|  | EIF4EBP1 | 0,1789 | 1,1959 | 0,2344 | 1,0000 |
|  | CXCL1 | 0,2264 | 1,2540 | 0,2537 | 1,0000 |
|  | TNF | -0,3706 | 0,6903 | 0,2630 | 1,0000 |
|  | IL6 | 0,1624 | 1,1764 | 0,2719 | 1,0000 |
|  | DNER | 0,6574 | 1,9298 | 0,2886 | 1,0000 |
|  | CD6 | 0,3001 | 1,3500 | 0,3026 | 1,0000 |
|  | IFNG | 0,7950 | 2,2144 | 0,3049 | 1,0000 |
|  | TSLP | -0,2845 | 0,7524 | 0,3268 | 1,0000 |
|  | IL20 | 0,2139 | 1,2385 | 0,3346 | 1,0000 |
|  | CXCL5 | 0,1300 | 1,1388 | 0,3633 | 1,0000 |
|  | IL5 | 0,0789 | 1,0821 | 0,3774 | 1,0000 |
|  | CCL19 | 0,1391 | 1,1492 | 0,4047 | 1,0000 |
|  | CD8A | 0,1657 | 1,1802 | 0,4054 | 1,0000 |
|  | CDCP1 | 0,1929 | 1,2128 | 0,4214 | 1,0000 |
|  | IL2 | 0,5796 | 1,7854 | 0,4403 | 1,0000 |
|  | LIF | 0,1806 | 1,1979 | 0,4465 | 1,0000 |
|  | TNFSF11 | -0,1764 | 0,8383 | 0,4700 | 1,0000 |
|  | CST5 | 0,1737 | 1,1896 | 0,4849 | 1,0000 |
|  | CASP8 | 0,1361 | 1,1458 | 0,4956 | 1,0000 |
|  | IL18R1 | 0,2184 | 1,2440 | 0,5187 | 1,0000 |
|  | ADA | -0,2091 | 0,8113 | 0,5371 | 1,0000 |
|  | FLT3LG | -0,1791 | 0,8360 | 0,5448 | 1,0000 |
|  | CCL23 | 0,1770 | 1,1937 | 0,5851 | 1,0000 |
|  | TNFSF12 | 0,2415 | 1,2731 | 0,6022 | 1,0000 |
|  | ARTN | 0,0985 | 1,1035 | 0,6071 | 1,0000 |
|  | LTA | 0,1624 | 1,1763 | 0,6163 | 1,0000 |
|  | IL1A | 0,1080 | 1,1141 | 0,6370 | 1,0000 |
|  | IL18 | 0,1094 | 1,1156 | 0,6487 | 1,0000 |
|  | SLAMF1 | 0,1234 | 1,1313 | 0,6491 | 1,0000 |
|  | CCL7 | 0,0949 | 1,0995 | 0,6785 | 1,0000 |
|  | NGF | 0,1447 | 1,1556 | 0,7650 | 1,0000 |
|  | LIFR | 0,1540 | 1,1665 | 0,7765 | 1,0000 |
|  | CCL4 | 0,0526 | 1,0540 | 0,8030 | 1,0000 |
|  | SULT1A1 | 0,0372 | 1,0379 | 0,8087 | 1,0000 |
|  | IL33 | 0,0685 | 1,0709 | 0,8366 | 1,0000 |
|  | IL2RB | 0,0448 | 1,0458 | 0,8506 | 1,0000 |
|  | IL22RA1 | 0,0440 | 1,0450 | 0,8509 | 1,0000 |
|  | IL20RA | -0,0412 | 0,9596 | 0,8537 | 1,0000 |
|  | IL13 | 0,0207 | 1,0209 | 0,8705 | 1,0000 |
|  | IL12B | 0,0292 | 1,0296 | 0,8874 | 1,0000 |
|  | FGF19 | 0,0180 | 1,0181 | 0,8978 | 1,0000 |
|  | IL4 | 0,0216 | 1,0219 | 0,9086 | 1,0000 |

Table S2: Results of the sensitivity analysis. The crude logistic model was calculated excluding all values below the lower limit of detection.

| Panel | Protein | Number observations | Model coefficient | Odds ratio | raw p-value | P-value adjusted by Yekutieli method |
| --- | --- | --- | --- | --- | --- | --- |
| CVDII | PTX3 | 611 | 1,1073 | 3,0262 | 0,0008 | 0,3860 |
|  | TNFRSF10A | 611 | 0,8393 | 2,3147 | 0,0063 | 1,0000 |
|  | VEGFD | 611 | 1,1146 | 3,0482 | 0,0100 | 1,0000 |
|  | LPL | 611 | 0,9002 | 2,4601 | 0,0115 | 1,0000 |
|  | FGF23 | 611 | 0,4361 | 1,5466 | 0,0120 | 1,0000 |
|  | TNFRSF10B | 611 | 0,6791 | 1,9721 | 0,0130 | 1,0000 |
|  | VSIG2 | 611 | 0,5086 | 1,6630 | 0,0162 | 1,0000 |
|  | XCL1 | 611 | 0,5532 | 1,7388 | 0,0188 | 1,0000 |
|  | CTRC | 611 | -0,3516 | 0,7035 | 0,0196 | 1,0000 |
|  | CCL17 | 611 | 0,3162 | 1,3719 | 0,0226 | 1,0000 |
|  | MMP12 | 611 | 0,4040 | 1,4978 | 0,0247 | 1,0000 |
|  | KITLG | 611 | -0,8418 | 0,4309 | 0,0276 | 1,0000 |
|  | IL27 | 611 | 0,8113 | 2,2508 | 0,0310 | 1,0000 |
|  | OLR1 | 611 | 0,4636 | 1,5898 | 0,0341 | 1,0000 |
|  | GH1 | 611 | 0,1628 | 1,1768 | 0,0366 | 1,0000 |
|  | OSCAR | 611 | 1,2689 | 3,5569 | 0,0388 | 1,0000 |
|  | MMP7 | 611 | 0,6499 | 1,9153 | 0,0447 | 1,0000 |
|  | FABP6 | 610 | 0,3313 | 1,3928 | 0,0451 | 1,0000 |
|  | ANGPT1 | 611 | 0,3017 | 1,3522 | 0,0467 | 1,0000 |
|  | FGF21 | 611 | 0,1969 | 1,2176 | 0,0500 | 1,0000 |
|  | HBEGF | 611 | 0,3801 | 1,4624 | 0,0512 | 1,0000 |
|  | IDUA | 611 | 0,5865 | 1,7976 | 0,0513 | 1,0000 |
|  | IL17D | 611 | 0,7984 | 2,2219 | 0,0535 | 1,0000 |
|  | PDGFB | 611 | 0,3135 | 1,3682 | 0,0662 | 1,0000 |
|  | NPPB | 549 | 0,1662 | 1,1809 | 0,0669 | 1,0000 |
|  | SORT1 | 611 | 0,7634 | 2,1456 | 0,0821 | 1,0000 |
|  | THPO | 611 | 0,5360 | 1,7091 | 0,0874 | 1,0000 |
|  | LEP | 611 | -0,2031 | 0,8162 | 0,0930 | 1,0000 |
|  | BOC | 610 | 0,8945 | 2,4460 | 0,0954 | 1,0000 |
|  | MERTK | 611 | -0,6605 | 0,5166 | 0,0971 | 1,0000 |
|  | SPON2 | 611 | 1,2607 | 3,5277 | 0,0985 | 1,0000 |
|  | DKK1 | 611 | 0,3360 | 1,3994 | 0,1071 | 1,0000 |
|  | GLO1 | 611 | 0,2720 | 1,3126 | 0,1184 | 1,0000 |
|  | F3 | 611 | 0,6650 | 1,9444 | 0,1207 | 1,0000 |
|  | SLAMF7 | 610 | 0,2899 | 1,3364 | 0,1515 | 1,0000 |
|  | AGRP | 611 | 0,4925 | 1,6364 | 0,1734 | 1,0000 |
|  | CTSL | 611 | 0,5556 | 1,7430 | 0,1749 | 1,0000 |
|  | THBS2 | 611 | 0,7622 | 2,1430 | 0,2060 | 1,0000 |
|  | CEACAM8 | 611 | 0,2572 | 1,2933 | 0,2117 | 1,0000 |
|  | FABP2 | 611 | 0,2035 | 1,2257 | 0,2120 | 1,0000 |
|  | CD84 | 611 | 0,4033 | 1,4968 | 0,2170 | 1,0000 |
|  | TNFRSF13B | 611 | 0,3373 | 1,4011 | 0,2274 | 1,0000 |
|  | DCN | 611 | -0,5655 | 0,5681 | 0,2322 | 1,0000 |
|  | TNFRSF11A | 611 | 0,2933 | 1,3408 | 0,2406 | 1,0000 |
|  | IL6 | 611 | 0,1739 | 1,1900 | 0,2450 | 1,0000 |
|  | CD40LG | 610 | 0,1356 | 1,1452 | 0,2603 | 1,0000 |
|  | HAO1 | 611 | -0,1253 | 0,8822 | 0,2614 | 1,0000 |
|  | ITGB1BP2 | 598 | 0,1297 | 1,1385 | 0,2666 | 1,0000 |
|  | PAPPA | 611 | 0,2574 | 1,2936 | 0,2841 | 1,0000 |
|  | IKBKG | 611 | 0,1229 | 1,1308 | 0,3051 | 1,0000 |
|  | HAVCR1 | 611 | 0,1740 | 1,1901 | 0,3062 | 1,0000 |
|  | DECR1 | 611 | 0,1001 | 1,1053 | 0,3114 | 1,0000 |
|  | CCL3 | 611 | 0,1999 | 1,2212 | 0,3153 | 1,0000 |
|  | CXCL1 | 611 | 0,1917 | 1,2113 | 0,3220 | 1,0000 |
|  | FCGR2B | 611 | 0,1651 | 1,1795 | 0,3540 | 1,0000 |
|  | PRSS8 | 611 | -0,2974 | 0,7428 | 0,3900 | 1,0000 |
|  | PRELP | 611 | 0,5574 | 1,7461 | 0,4121 | 1,0000 |
|  | HSPB1 | 611 | -0,3087 | 0,7344 | 0,4206 | 1,0000 |
|  | IL1RN | 611 | -0,2096 | 0,8109 | 0,4219 | 1,0000 |
|  | ADAMTS13 | 610 | 0,7294 | 2,0737 | 0,4321 | 1,0000 |
|  | ADM | 611 | 0,2344 | 1,2642 | 0,4601 | 1,0000 |
|  | IL4R | 611 | 0,2817 | 1,3254 | 0,4638 | 1,0000 |
|  | PIGR | 611 | 0,6918 | 1,9973 | 0,4640 | 1,0000 |
|  | PARP1 | 611 | 0,1415 | 1,1520 | 0,4781 | 1,0000 |
|  | IL16 | 611 | 0,1926 | 1,2124 | 0,4840 | 1,0000 |
|  | SERPINA12 | 598 | -0,0921 | 0,9121 | 0,4971 | 1,0000 |
|  | LGALS9 | 611 | 0,2770 | 1,3192 | 0,5065 | 1,0000 |
|  | THBD | 611 | 0,2687 | 1,3083 | 0,5123 | 1,0000 |
|  | SRC | 611 | -0,1457 | 0,8644 | 0,5270 | 1,0000 |
|  | MARCO | 611 | 0,3758 | 1,4561 | 0,5615 | 1,0000 |
|  | AGER | 611 | 0,1718 | 1,1874 | 0,5664 | 1,0000 |
|  | CBLIF | 610 | -0,0719 | 0,9306 | 0,5695 | 1,0000 |
|  | GDF2 | 611 | 0,2214 | 1,2478 | 0,5695 | 1,0000 |
|  | AMBP | 611 | 0,3809 | 1,4636 | 0,5973 | 1,0000 |
|  | REN | 611 | 0,0640 | 1,0660 | 0,6586 | 1,0000 |
|  | SELPLG | 611 | 0,2144 | 1,2391 | 0,6712 | 1,0000 |
|  | IL18 | 611 | 0,1003 | 1,1056 | 0,6788 | 1,0000 |
|  | PRSS27 | 611 | 0,1066 | 1,1125 | 0,7305 | 1,0000 |
|  | BMP6 | 611 | 0,0890 | 1,0930 | 0,7589 | 1,0000 |
|  | PGF | 610 | 0,1134 | 1,1200 | 0,7616 | 1,0000 |
|  | CD4 | 611 | 0,1114 | 1,1179 | 0,7912 | 1,0000 |
|  | HMOX1 | 611 | -0,0926 | 0,9116 | 0,8027 | 1,0000 |
|  | STK4 | 609 | 0,0377 | 1,0384 | 0,8082 | 1,0000 |
|  | IL1RL2 | 611 | 0,0742 | 1,0771 | 0,8305 | 1,0000 |
|  | TGM2 | 611 | 0,0528 | 1,0542 | 0,8376 | 1,0000 |
|  | CA5A | 532 | 0,0419 | 1,0428 | 0,8429 | 1,0000 |
|  | PDCD1LG2 | 611 | 0,0611 | 1,0630 | 0,8881 | 1,0000 |
|  | SOD2 | 611 | -0,1479 | 0,8625 | 0,9011 | 1,0000 |
|  | ACE2 | 611 | -0,0073 | 0,9927 | 0,9755 | 1,0000 |
|  | F2R | 611 | -0,0101 | 0,9900 | 0,9804 | 1,0000 |
|  | TEK | 611 | 0,0110 | 1,0110 | 0,9852 | 1,0000 |
|  | FST | 611 | -0,0043 | 0,9957 | 0,9897 | 1,0000 |
|  |  |  |  |  |  |  |
| CVD III | PLAUR | 611 | 1,4756 | 4,3738 | 0,0000 | 0,0165 |
|  | SCGB3A2 | 611 | 0,6116 | 1,8434 | 0,0007 | 0,1569 |
|  | TNFSF13B | 611 | 1,1187 | 3,0609 | 0,0012 | 0,1822 |
|  | TFF3 | 611 | 0,7354 | 2,0863 | 0,0017 | 0,1822 |
|  | IGFBP2 | 611 | 0,7619 | 2,1423 | 0,0019 | 0,1822 |
|  | IL18BP | 611 | 1,1169 | 3,0553 | 0,0029 | 0,2301 |
|  | IGFBP1 | 608 | 0,3622 | 1,4365 | 0,0072 | 0,3814 |
|  | LGALS4 | 611 | 0,6163 | 1,8521 | 0,0077 | 0,3814 |
|  | SPP1 | 611 | 0,6978 | 2,0093 | 0,0088 | 0,3814 |
|  | SFTPD | 610 | 0,4707 | 1,6011 | 0,0091 | 0,3814 |
|  | COL1A1 | 611 | 0,9797 | 2,6637 | 0,0091 | 0,3814 |
|  | F11R | 611 | 0,2691 | 1,3087 | 0,0098 | 0,3814 |
|  | IL2RA | 611 | 0,7528 | 2,1229 | 0,0113 | 0,3814 |
|  | TNFRSF14 | 611 | 0,5855 | 1,7959 | 0,0114 | 0,3814 |
|  | GDF15 | 611 | 0,5257 | 1,6916 | 0,0128 | 0,3872 |
|  | GP6 | 611 | 0,5632 | 1,7564 | 0,0132 | 0,3872 |
|  | PECAM1 | 611 | 0,3600 | 1,4333 | 0,0155 | 0,4276 |
|  | PDGFA | 611 | 0,3883 | 1,4745 | 0,0180 | 0,4700 |
|  | SELP | 611 | 0,3688 | 1,4459 | 0,0225 | 0,5565 |
|  | TNFRSF1B | 611 | 0,6006 | 1,8232 | 0,0283 | 0,6428 |
|  | CCL15 | 611 | 0,5012 | 1,6507 | 0,0287 | 0,6428 |
|  | TNFRSF11B | 611 | 0,9084 | 2,4802 | 0,0326 | 0,6766 |
|  | TNFRSF10C | 611 | 0,5951 | 1,8132 | 0,0331 | 0,6766 |
|  | MMP9 | 611 | 0,4052 | 1,4996 | 0,0362 | 0,7084 |
|  | IL1R1 | 611 | 0,9245 | 2,5207 | 0,0414 | 0,7770 |
|  | CXCL16 | 611 | 0,8136 | 2,2560 | 0,0470 | 0,8241 |
|  | PGLYRP1 | 611 | 0,5667 | 1,7624 | 0,0474 | 0,8241 |
|  | TIMP4 | 611 | 0,5814 | 1,7885 | 0,0514 | 0,8621 |
|  | KLK6 | 611 | 0,6400 | 1,8964 | 0,0564 | 0,9133 |
|  | ALCAM | 611 | 0,9409 | 2,5623 | 0,0722 | 1,0000 |
|  | TFPI | 611 | 0,7395 | 2,0950 | 0,0788 | 1,0000 |
|  | IGFBP7 | 611 | 0,6056 | 1,8323 | 0,0856 | 1,0000 |
|  | PRTN3 | 611 | 0,3990 | 1,4903 | 0,0936 | 1,0000 |
|  | TNFRSF1A | 611 | 0,4466 | 1,5630 | 0,0956 | 1,0000 |
|  | CHIT1 | 596 | 0,2491 | 1,2829 | 0,0965 | 1,0000 |
|  | SPON1 | 608 | 0,7601 | 2,1386 | 0,1017 | 1,0000 |
|  | AXL | 611 | 0,6415 | 1,8993 | 0,1106 | 1,0000 |
|  | CTSZ | 610 | 0,5232 | 1,6873 | 0,1262 | 1,0000 |
|  | TREML2 | 611 | 0,4322 | 1,5406 | 0,1293 | 1,0000 |
|  | SERPINE1 | 611 | 0,2463 | 1,2792 | 0,1398 | 1,0000 |
|  | VWF | 611 | 0,2071 | 1,2301 | 0,1449 | 1,0000 |
|  | LTBR | 611 | 0,4872 | 1,6277 | 0,1566 | 1,0000 |
|  | CD93 | 611 | 0,6010 | 1,8239 | 0,1568 | 1,0000 |
|  | PLAU | 611 | 0,4322 | 1,5407 | 0,1671 | 1,0000 |
|  | CPA1 | 611 | -0,2399 | 0,7867 | 0,1698 | 1,0000 |
|  | CPB1 | 611 | -0,2368 | 0,7892 | 0,1812 | 1,0000 |
|  | CHI3L1 | 611 | 0,1833 | 1,2012 | 0,1832 | 1,0000 |
|  | CASP3 | 611 | 0,1733 | 1,1892 | 0,1838 | 1,0000 |
|  | MPO | 611 | 0,4585 | 1,5817 | 0,1842 | 1,0000 |
|  | RARRES2 | 611 | 0,6691 | 1,9524 | 0,1960 | 1,0000 |
|  | CTSD | 611 | 0,3155 | 1,3710 | 0,2103 | 1,0000 |
|  | IL17RA | 611 | 0,3135 | 1,3683 | 0,2138 | 1,0000 |
|  | ICAM2 | 611 | 0,4492 | 1,5671 | 0,2183 | 1,0000 |
|  | MMP2 | 611 | 0,4735 | 1,6055 | 0,2274 | 1,0000 |
|  | PCSK9 | 611 | 0,4697 | 1,5994 | 0,2301 | 1,0000 |
|  | MMP3 | 611 | 0,2246 | 1,2519 | 0,2598 | 1,0000 |
|  | EPHB4 | 611 | 0,4256 | 1,5305 | 0,2624 | 1,0000 |
|  | CSTB | 611 | 0,2467 | 1,2798 | 0,2695 | 1,0000 |
|  | CCL24 | 611 | 0,1641 | 1,1784 | 0,2754 | 1,0000 |
|  | CCL16 | 611 | 0,2788 | 1,3215 | 0,2760 | 1,0000 |
|  | FAS | 611 | 0,2737 | 1,3149 | 0,3100 | 1,0000 |
|  | ACP5 | 611 | 0,3512 | 1,4207 | 0,3216 | 1,0000 |
|  | LGALS3 | 611 | 0,3297 | 1,3906 | 0,3676 | 1,0000 |
|  | SIRPA | 611 | 0,2623 | 1,2999 | 0,4177 | 1,0000 |
|  | NOTCH3 | 611 | 0,2983 | 1,3476 | 0,4224 | 1,0000 |
|  | IL6R | 611 | 0,2788 | 1,3215 | 0,4299 | 1,0000 |
|  | IL1RL1 | 611 | 0,1826 | 1,2003 | 0,4615 | 1,0000 |
|  | HSPG2 | 611 | 0,3096 | 1,3629 | 0,4795 | 1,0000 |
|  | NPPB | 610 | 0,0729 | 1,0757 | 0,5091 | 1,0000 |
|  | FABP4 | 611 | 0,1128 | 1,1194 | 0,5122 | 1,0000 |
|  | ITGB2 | 611 | -0,2531 | 0,7764 | 0,5197 | 1,0000 |
|  | CD163 | 611 | 0,1905 | 1,2098 | 0,5241 | 1,0000 |
|  | EGFR | 611 | -0,3493 | 0,7052 | 0,5754 | 1,0000 |
|  | DLK1 | 611 | 0,1151 | 1,1220 | 0,5962 | 1,0000 |
|  | PON3 | 611 | 0,1147 | 1,1215 | 0,6026 | 1,0000 |
|  | LDLR | 611 | -0,1266 | 0,8811 | 0,6178 | 1,0000 |
|  | EPCAM | 611 | 0,0544 | 1,0559 | 0,6602 | 1,0000 |
|  | RETN | 611 | 0,1180 | 1,1253 | 0,6711 | 1,0000 |
|  | TFRC | 611 | -0,1030 | 0,9021 | 0,6990 | 1,0000 |
|  | ANPEP | 611 | -0,1778 | 0,8371 | 0,7034 | 1,0000 |
|  | CNTN1 | 611 | -0,1492 | 0,8614 | 0,7069 | 1,0000 |
|  | BLMH | 611 | 0,0892 | 1,0933 | 0,7328 | 1,0000 |
|  | CCL2 | 611 | 0,0731 | 1,0759 | 0,7516 | 1,0000 |
|  | MB | 611 | 0,0632 | 1,0652 | 0,7559 | 1,0000 |
|  | PI3 | 611 | -0,0446 | 0,9564 | 0,8203 | 1,0000 |
|  | PLAT | 611 | 0,0480 | 1,0491 | 0,8241 | 1,0000 |
|  | CDH5 | 611 | -0,0724 | 0,9302 | 0,8614 | 1,0000 |
|  | AZU1 | 609 | 0,0230 | 1,0232 | 0,8705 | 1,0000 |
|  | MEPE | 611 | 0,0301 | 1,0305 | 0,9186 | 1,0000 |
|  | IL1R2 | 611 | 0,0291 | 1,0296 | 0,9435 | 1,0000 |
|  | GRN | 611 | 0,0309 | 1,0314 | 0,9471 | 1,0000 |
|  | SELE | 611 | -0,0112 | 0,9889 | 0,9607 | 1,0000 |
|  |  |  |  |  |  |  |
| Inflammation | MMP1 | 611 | 0,4375 | 1,5489 | 0,0009 | 0,2571 |
|  | CXCL9 | 611 | 0,4647 | 1,5915 | 0,0014 | 0,2571 |
|  | CXCL11 | 611 | 0,4783 | 1,6134 | 0,0017 | 0,2571 |
|  | VEGFA | 611 | 0,9260 | 2,5244 | 0,0029 | 0,3239 |
|  | IL15RA | 576 | 1,3693 | 3,9328 | 0,0067 | 0,5266 |
|  | CCL13 | 611 | 0,5833 | 1,7920 | 0,0071 | 0,5266 |
|  | FGF23 | 611 | 0,4414 | 1,5549 | 0,0084 | 0,5341 |
|  | OSM | 611 | 0,4694 | 1,5991 | 0,0099 | 0,5500 |
|  | TNFRSF9 | 611 | 0,6194 | 1,8578 | 0,0112 | 0,5528 |
|  | MMP10 | 611 | 0,5261 | 1,6924 | 0,0188 | 0,7055 |
|  | TGFA | 611 | 0,9247 | 2,5212 | 0,0203 | 0,7055 |
|  | CCL25 | 611 | 0,6046 | 1,8305 | 0,0208 | 0,7055 |
|  | TGFB1 | 611 | 0,6182 | 1,8555 | 0,0230 | 0,7055 |
|  | CCL11 | 611 | 0,7431 | 2,1025 | 0,0230 | 0,7055 |
|  | TNFSF14 | 611 | 0,4871 | 1,6277 | 0,0241 | 0,7055 |
|  | CXCL6 | 611 | 0,3770 | 1,4579 | 0,0253 | 0,7055 |
|  | IL17A | 518 | 0,3173 | 1,3734 | 0,0384 | 0,9759 |
|  | S100A12 | 611 | 0,3113 | 1,3652 | 0,0403 | 0,9759 |
|  | CD244 | 611 | 0,5680 | 1,7648 | 0,0416 | 0,9759 |
|  | KITLG | 611 | -0,7867 | 0,4553 | 0,0503 | 1,0000 |
|  | CXCL10 | 611 | 0,2838 | 1,3281 | 0,0508 | 1,0000 |
|  | CD5 | 611 | 0,7084 | 2,0307 | 0,0539 | 1,0000 |
|  | FGF21 | 611 | 0,2086 | 1,2319 | 0,0563 | 1,0000 |
|  | CCL20 | 611 | 0,2401 | 1,2714 | 0,0595 | 1,0000 |
|  | CSF1 | 611 | 1,2471 | 3,4801 | 0,0612 | 1,0000 |
|  | STAMBP | 611 | 0,2322 | 1,2614 | 0,0632 | 1,0000 |
|  | NTF3 | 611 | 0,4568 | 1,5790 | 0,0728 | 1,0000 |
|  | TNFRSF11B | 611 | 0,7635 | 2,1458 | 0,0754 | 1,0000 |
|  | CX3CL1 | 611 | 0,5663 | 1,7618 | 0,0773 | 1,0000 |
|  | AXIN1 | 611 | 0,1859 | 1,2043 | 0,0784 | 1,0000 |
|  | GDNF | 610 | 0,5311 | 1,7008 | 0,0797 | 1,0000 |
|  | IL7 | 611 | 0,3543 | 1,4251 | 0,0820 | 1,0000 |
|  | NRTN | 99 | 0,6003 | 1,8228 | 0,0832 | 1,0000 |
|  | CCL8 | 611 | 0,3811 | 1,4638 | 0,0970 | 1,0000 |
|  | IL10RB | 611 | 0,7458 | 2,1082 | 0,0992 | 1,0000 |
|  | CD40 | 611 | 0,4271 | 1,5328 | 0,1041 | 1,0000 |
|  | CCL3 | 611 | 0,3028 | 1,3536 | 0,1083 | 1,0000 |
|  | CCL28 | 611 | 0,6405 | 1,8975 | 0,1134 | 1,0000 |
|  | CXCL8 | 611 | 0,2657 | 1,3043 | 0,1360 | 1,0000 |
|  | IL24 | 201 | 0,2429 | 1,2750 | 0,1507 | 1,0000 |
|  | CCL2 | 611 | 0,4458 | 1,5617 | 0,1536 | 1,0000 |
|  | IL10 | 611 | 0,3469 | 1,4146 | 0,1700 | 1,0000 |
|  | IL4 | 86 | -0,9549 | 0,3848 | 0,1752 | 1,0000 |
|  | HGF | 611 | 0,4903 | 1,6329 | 0,1761 | 1,0000 |
|  | SIRT2 | 608 | 0,1524 | 1,1647 | 0,1835 | 1,0000 |
|  | PLAU | 611 | 0,5594 | 1,7497 | 0,1901 | 1,0000 |
|  | TNFSF10 | 611 | 0,6363 | 1,8894 | 0,1906 | 1,0000 |
|  | IL2RB | 99 | -0,8477 | 0,4284 | 0,1964 | 1,0000 |
|  | IL33 | 26 | -3,7793 | 0,0228 | 0,2147 | 1,0000 |
|  | CD274 | 611 | 0,3281 | 1,3884 | 0,2309 | 1,0000 |
|  | EIF4EBP1 | 611 | 0,1789 | 1,1959 | 0,2344 | 1,0000 |
|  | CXCL1 | 611 | 0,2264 | 1,2540 | 0,2537 | 1,0000 |
|  | IL6 | 611 | 0,1624 | 1,1764 | 0,2719 | 1,0000 |
|  | IL10RA | 412 | 0,1670 | 1,1818 | 0,2735 | 1,0000 |
|  | ARTN | 319 | -0,3224 | 0,7244 | 0,2874 | 1,0000 |
|  | DNER | 611 | 0,6574 | 1,9298 | 0,2886 | 1,0000 |
|  | IL20 | 81 | 0,2902 | 1,3367 | 0,2968 | 1,0000 |
|  | CD6 | 611 | 0,3001 | 1,3500 | 0,3026 | 1,0000 |
|  | IL22RA1 | 62 | -1,9820 | 0,1378 | 0,3054 | 1,0000 |
|  | TSLP | 76 | -1,3851 | 0,2503 | 0,3067 | 1,0000 |
|  | CXCL5 | 611 | 0,1300 | 1,1388 | 0,3633 | 1,0000 |
|  | CCL19 | 611 | 0,1391 | 1,1492 | 0,4047 | 1,0000 |
|  | CD8A | 611 | 0,1657 | 1,1802 | 0,4054 | 1,0000 |
|  | CDCP1 | 611 | 0,1929 | 1,2128 | 0,4214 | 1,0000 |
|  | TNFSF11 | 611 | -0,1764 | 0,8383 | 0,4700 | 1,0000 |
|  | CST5 | 611 | 0,1737 | 1,1896 | 0,4849 | 1,0000 |
|  | CASP8 | 611 | 0,1361 | 1,1458 | 0,4956 | 1,0000 |
|  | IL18R1 | 611 | 0,2184 | 1,2440 | 0,5187 | 1,0000 |
|  | ADA | 611 | -0,2091 | 0,8113 | 0,5371 | 1,0000 |
|  | FLT3LG | 611 | -0,1791 | 0,8360 | 0,5448 | 1,0000 |
|  | TNF | 54 | -0,4005 | 0,6700 | 0,5724 | 1,0000 |
|  | CCL23 | 611 | 0,1770 | 1,1937 | 0,5851 | 1,0000 |
|  | TNFSF12 | 611 | 0,2415 | 1,2731 | 0,6022 | 1,0000 |
|  | IL1A | 48 | 0,1829 | 1,2007 | 0,6124 | 1,0000 |
|  | IL13 | 150 | -0,1180 | 0,8887 | 0,6129 | 1,0000 |
|  | LTA | 611 | 0,1624 | 1,1763 | 0,6163 | 1,0000 |
|  | CCL7 | 581 | 0,1137 | 1,1205 | 0,6374 | 1,0000 |
|  | FGF5 | 377 | 0,3400 | 1,4049 | 0,6393 | 1,0000 |
|  | IL18 | 611 | 0,1094 | 1,1156 | 0,6487 | 1,0000 |
|  | SLAMF1 | 611 | 0,1234 | 1,1313 | 0,6491 | 1,0000 |
|  | NGF | 611 | 0,1447 | 1,1556 | 0,7650 | 1,0000 |
|  | LIFR | 611 | 0,1540 | 1,1665 | 0,7765 | 1,0000 |
|  | CCL4 | 611 | 0,0526 | 1,0540 | 0,8030 | 1,0000 |
|  | IL5 | 219 | -0,0294 | 0,9711 | 0,8210 | 1,0000 |
|  | SULT1A1 | 609 | 0,0237 | 1,0240 | 0,8794 | 1,0000 |
|  | IL12B | 611 | 0,0292 | 1,0296 | 0,8874 | 1,0000 |
|  | FGF19 | 611 | 0,0180 | 1,0181 | 0,8978 | 1,0000 |
|  | LIF | 59 | 0,0025 | 1,0025 | 0,9960 | 1,0000 |
|  |  |  |  |  |  |  |
